# Construction of Chiral Cyclic Compounds Enabled by Enantioselective Photocatalysis

**DOI:** 10.3390/molecules27020359

**Published:** 2022-01-07

**Authors:** Bo Xu, Xiaotian Shi, Xiang Liu, Hua Cao

**Affiliations:** Guangdong Cosmetics Engineering & Technology Research Center, School of Chemistry and Chemical Engineering, Guangdong Pharmaceutical University, Zhongshan 528458, China; xajaken@126.com (B.X.); sxtgoo@gmail.com (X.S.)

**Keywords:** photocatalysis, chiral cyclic compounds, asymmetric catalysis, enantioselectivity

## Abstract

Chiral cyclic molecules are some of the most important compounds in nature, and are widely used in the fields of drugs, materials, synthesis, etc. Enantioselective photocatalysis has become a powerful tool for organic synthesis of chiral cyclic molecules. Herein, this review summarized the research progress in the synthesis of chiral cyclic compounds by photocatalytic cycloaddition reaction in the past 5 years, and expounded the reaction conditions, characters, and corresponding proposed mechanism, hoping to guide and promote the development of this field.

## 1. Introduction

In recent years, enantioselective photocatalysis has been successfully applied to extensive practical work of organic synthesis [1,2,3], providing an alternative method for the production of valuable chiral molecules. In this regard, many chemists such as Yoon, MacMillan, Bach, etc., made great contributions to the development of milder and more efficient enantioselective cycloaddition reaction through the organic photocatalysis. List and MacMillan were awarded the 2021 Nobel prize in chemistry for their “development in asymmetric organocatalysis”.

This review mainly discusses the research progress of enantioselective photocatalysis for constructing chiral cyclic compounds by photo-induced asymmetric cycloaddition reaction in the past 5 years, though some comprehensive contents regarding this topic were reported by the pioneers [4,5,6,7,8]. This paper is divided into seven parts according to the structural types of rings: the construction of 3-membered rings, 4-membered rings, 5-membered rings, 6-membered rings, 7-membered rings, macroring, and multi-rings. In contrast, there are significantly more reports about the construction of 4-membered rings via enantioselective photocatalysis. In these transformations, the use of chiral catalyst could furnish an appropriate chiral environment and improve the photocycloaddition enantioselectivity; some of them have become the representative chiral photocatalyst for enantioselective photocatalysis, such as chiral oxazaborolidine Lewis acid, chiral thioxones, ruthenium catalysts, iridium catalysts, chiral amine catalysts, and chiral phosphoric acids. Especially in recent years, chiral organocatalysts are more and more widely used in enantioselective photocatalysis [9]. In addition, the catalytic system had also been developed from the original single catalysis system to the current double catalysis, or even triple catalysis, system.

## 2. Enantioselective Cycloaddition via Visible-Light Catalysis

### 2.1. Enantioselective Formation of 3-Membered Ring by Visible Light Catalysis

**Scheme 1:**
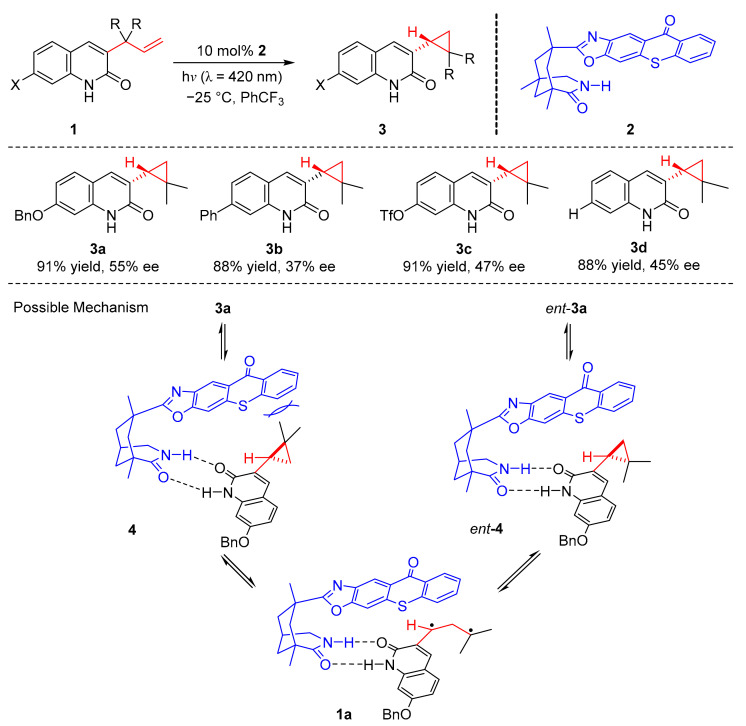
Enantioselective formation of 3-cyclopropyl-quinolones.

A ternary ring is a kind of tension ring. It is a very challenging process to construct chiral ternary ring molecules via photocatalysis. There are very few reports on the construction of 3-membered ring by enantioselective photocatalysis in the past five years. In 2019, Bach et al. reported a simple, efficient, and enantioselective route to obtain the cyclopropyl substituted quinolone compounds [10]. Under the irradiation of visible light (λ = 420 nm), the 3-allyl-substituted quinolones (**1**) underwent a triplet sensitized di-π-methane rearrangement reaction to form 3-cyclopropylquinolones (**3**) in the presence of a chiral hydrogen bonding sensitizer thioxanthone (**2**) (Scheme 1). The reaction showed excellent yields in most cases and moderate enantioselectivity (88–96% yield, 32–55% ee). The author proposed a mechanism as follows: (**1**) was associated with a chiral hydrogen bonding sensitizer (**2**) to form the 1,3-diradical intermediate (**1a**), which further closed the ring to form the complex (*ent*-**4**) or complex (**4**). Owing to the geometric constraints in complex (**1a**), generating (*ent*-**4**) is expected to decay preferentially. In other words, this process favours formation of (**4**) while the latter process shows a preference for *ent-***3a** (higher association constant Ka than [**3a**]), thus reducing the enantioselectivity of the deracemization process. Finally, the major enantiomer 3-cyclopropyl-quinolones (**3**) were obtained.

### 2.2. Enantioselective Formation of 4-Membered Ring by Visible Light Catalysis

**Scheme 2:**
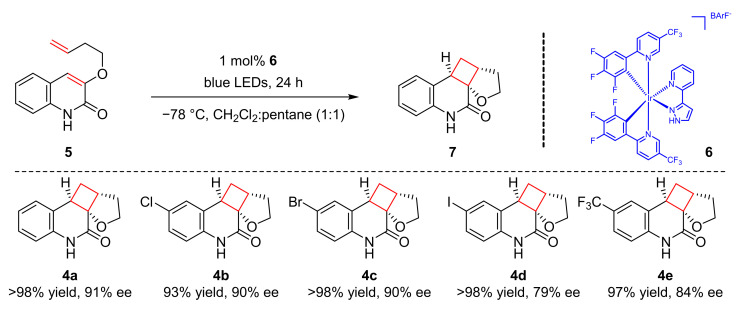
Enantioselective photocycloaddition of 3-alkoxyquinolones.

The technology for the construction of chiral 4-membered ring compounds by enantioselective photocatalysis has become more mature, and [2+2] photocycloaddition is the most common synthesis method. In 2017, an effective and enantioselectivie chiral iridium catalyzed [2+2] photocycloaddition was reported by Yoon et al. [11], who used structurally related 3-alkoxyquinolones (**5**) irradiated by blue LED light with Ir(III) photosensitizer (**6**) to synthesize products (**7**) in good yields and enantioselectivitiy (up to 98% yield, up to 91% ee) (Scheme 2). Chloro- and bromo-substituted quinolones performed well but iodinated substrate displayed lower enantioselectivity. The excellent performance is still capable of modified alkene moiety with small enantioselective decline.

**Scheme 3:**
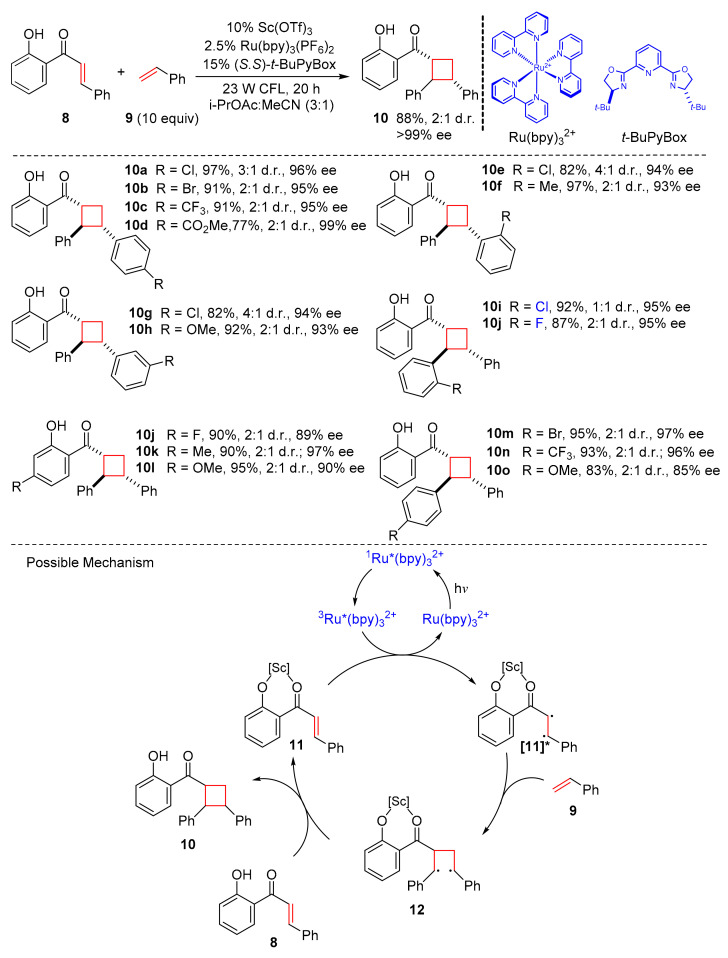
Enantioselective crossed photocycloadditions to synthesize unsymmetrical cyclobutanes.

Earlier, Yoon et al. developed a new strategy to achieve enantioselective [2+2] photocycloaddition of 2′-hydroxychalcones via Lewis acid-catalyzed triplet energy transfer [12]. Subsequently, they reported a chiral Lewis acid catalyzed triplet sensitization for enantioselective crossed photocycloaddition to synthesize highly enantioenriched cyclobutanes in 2017 [13]. In this work, 2′-hydroxychalcones (**8**) could couple with styrenes (**9**) to construct diarylcyclobutanes (**10**) in the presence of Sc(OTf)_3_, *t*-BuPybox, and Ru(bpy)_3_^2+^ upon the irradiation of 23 W CFL (Scheme 3). The transformations showed excellent yields and high enantioselectivity (up to 97% yield, up to 99% ee). The styrene ring could be substituted by a variety of electron-donating groups or electron-withdrawing groups, and the styryl double bond was also modified by some substituents with high ee. This method also provided a direct approach to the synthesis of diarylcyclobutane natural products, such as norlignan 3. The proposed mechanism was conducted as follows. 2′-hydroxychalcones (**8**) initially cooperated with Lewis acid to form the Lewis-acid-bound substrates (**11**), which could be transform into (11*) via triplet energy transfer by Ru(bpy)_3_^2+^ under the irradiation of 23 W CFL, then styrenes (**9**) captured with 1,4-diradical intermediates (**12**) to produce diarylcyclobutanes (**10**).

**Scheme 4:**
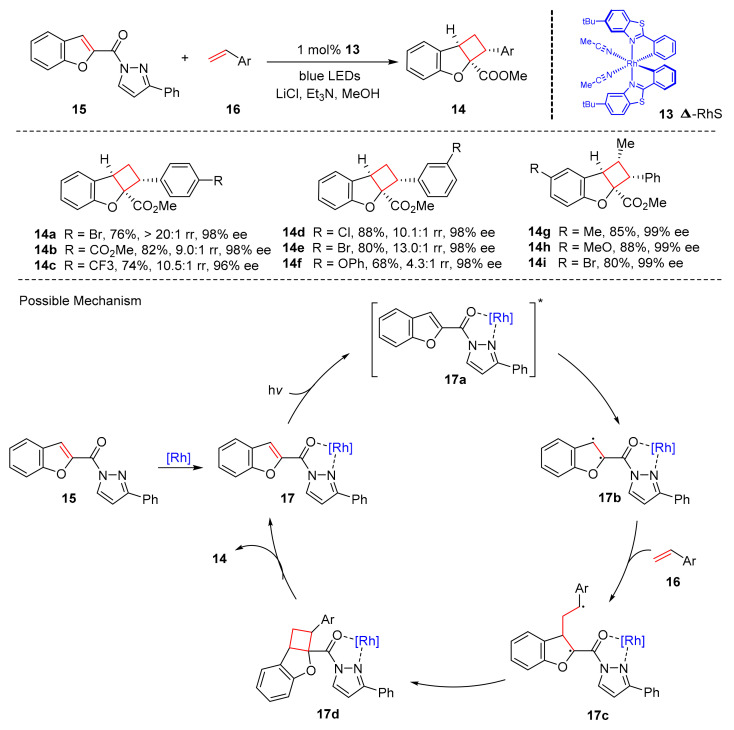
Catalytic asymmetric dearomatization by visible-light-activated [2+2] photocycloaddition.

In 2018, a new visible-light-activated [2+2] photocycloaddition to asymmetric dearomatization of benzofurans was reported by Meggers et al., using the chiral rhodium Lewis acid catalyst (**13**) to synthesize the photocycloaddition products (**14**) from benzofurans (**15**) and styrenes (**16**) upon the irradiation of bule light in good yields and excellent enantioselectivity (up to 91% yield, up to 99% ee) (Scheme 4) [14]. The phenyl group of styrenes substituted by halogen groups and bulky groups in *para*- and *meta*-position possess good tolerance but there are greater effects at *ortho*-position. The substituted benzene moiety of benzofurans could also be tolerated with (E)-β-methylstyrene and (E)-β-alkylstyrene. Moreover, the methodology could be applied to prepare benzothiophene as well. The proposed mechanism was conducted as follows. The first step was the cooperation of (**15**) with the chiral rhodium catalyst to form complex (**17**), then the complex (**17**) transformed into (**17a**) upon the irradiation of blue light, which subsequently underwent intersystem crossing to generate (**17b**). The intermediates (**17b**) further combined with alkenes (**16**) to give the 1,4-biradical intermediates (**17c**), which closed the ring to furnish the desirable photocycloaddition products (**14**).

**Scheme 5:**
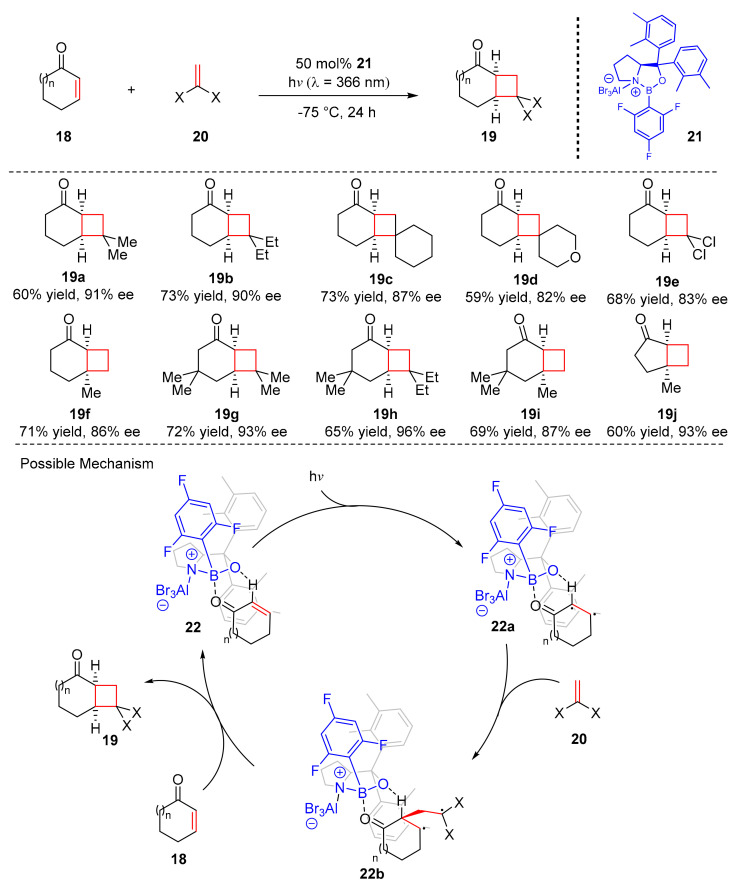
Enantioselective [2+2] photocycloaddition reaction of cyclic enones with 1,1-disubstituted olefins.

In 2018, Bach et al. found an enantioselective [2+2] photocycloaddition reaction of cyclic enones (**18**) to synthesize cyclobutanes (**19**) in good yields and enantioselectivity (up to 82% yield, up to 96% ee) with olefins (**20**) induced by visible light (λ = 366 nm) (Scheme 5) [15]. A new substituted chiral oxazoborolidine-AlBr_3_ Lewis complex (**21**) was used as the photocatalyst. 1,1-Disubstituted olefins were well tolerated. A variety of substituents at β-position of cyclic enones could also participate the reaction with high enantioselectivity. The proposed mechanism was conducted as follows. Initially, (**18**) cooperated with (**21**) to form the intermediates (**22**) which could be transformed into (**22a**), then (**22a**) reacted with olefins **20** to give relatively 1,4-diradical intermediates (**22b**), which released (**21**) to produce cyclization products (**19**).

**Scheme 6:**
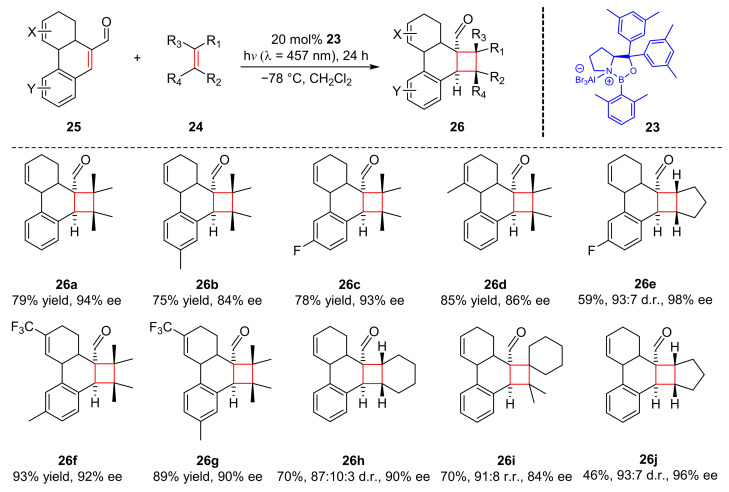
Chiral oxazaborolidine Lewis acid catalyzed photocycloaddition of olefins with phenanthrene-9-carboxaldehydes.

Another chiral oxazaborolidine Lewis acid (**23**) catalyzed photocycloaddition was reported by Bach et al. for the cooperation of olefins (**24**) with phenanthrene-9-carboxaldehydes (**25**) in 2018 (Scheme 6) [16]. The photocyclization products (**26**) were afforded with good enantioselectivity (46–93% yield, 82–98% ee) upon the irradiation of visible light (λ = 457 nm). Substituents at 3- and 6-positions of phenanthrene-9-carboxaldehydes were well tolerated, but the substituents at 2- and 5-positions could decrease the enantioselectivity somehow. A similar mechanism has been mentioned before (Scheme 5).

**Scheme 7:**
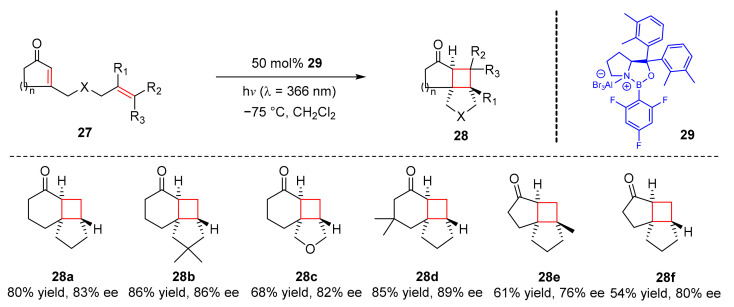
Intramolecular [2+2] photocycloaddition of 3-alkenyl- 2-cycloalkenones.

In 2019, Bach et al. reported an intramolecular [2+2] photocycloaddition of 3-alkenyl-2-cycloalkenones (**27**) to form photocyclization products (**28**) by utilizing a chiral oxazaborolidine Lewis acid catalyst (**29**) upon the irradiation of visible light (λ = 366 nm). The photocyclization products were produced well in yields and enantioselectivity (54–86% yield, 76–96% ee) (Scheme 7) [17]. Alkenyl chains substituted at 3-position of 2-cyclopentenones and 2-cyclohexenones were effective in this photocycloaddition. Moreover, the alkenyl chains could also be connected using oxygen atom.

**Scheme 8:**
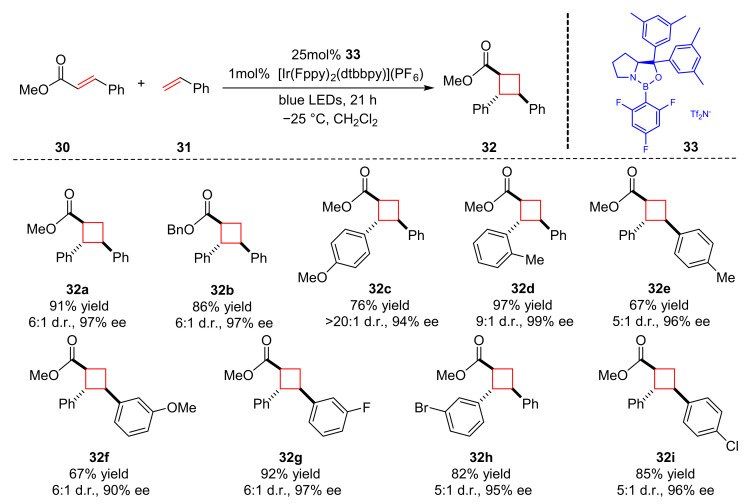
Enantioselective photocycloaddition reaction of cinnamate esters with styrenes.

On the basis of enantioselective Lewis acid catalyzed [2+2] photocycloaddition of 2′-hydroxychalcones [12], Yoon et al. reported the enantioselective photocycloaddition reaction of cinnamate esters (**30**) and styrenes (**31**) to synthesize the cyclobutane derivatives (**32**) upon the irradiation of blue LED light, using a new dual catalytic system by merging chiral oxazaborolidine Lewis acid (**33**) with Ir(III) photocatalyst in 2019 (Scheme 8) [18]. Electron-donating and electron-withdrawing substituents on the styrenes were well tolerated, a variety of β-aryl substituents of cinnamate esters could also react well in good yields and excellent enantioselectivity (67–97% yield, 92–98% ee). The proposed mechanism proceeds via a similar catalytic cycle, which has been mentioned before (Scheme 5).

**Scheme 9:**
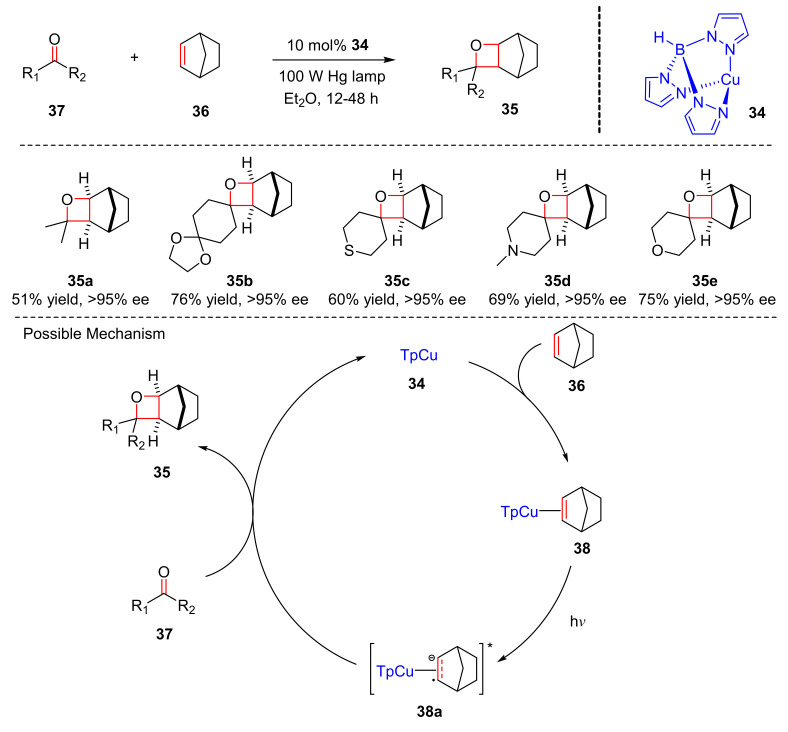
Intermolecular [2+2] carbonyl-olefin photocycloadditions enabled by TpCu.

In 2019, Schmidt et al. disclosed a Cu(I) (**34**) catalyzed carbonyl-olefin [2+2] photocycloaddition of carbonyl substrates (**36**) and olefins (**37**) to produce 4-membered ring products (**35**) with 100 W Hg lamp in good yields and excellent enantioselectivity (45–75% yield, >95% ee) (Scheme 9) [19]. Alkyl ketones were content substrates. Cyclic ketones which contain acetal, ether, basic tertiary amine, and thio-ether performed better enantioselectivity. The proposed mechanism began via activation of the alkene (**36**) to form the corresponding complex (**38**) with TpCu (**34**), which could be excited to generate (**38a**). Subsequently, (**38a**) reacted with olefins (**37**) to generate oxetanes (**35**).

**Scheme 10:**
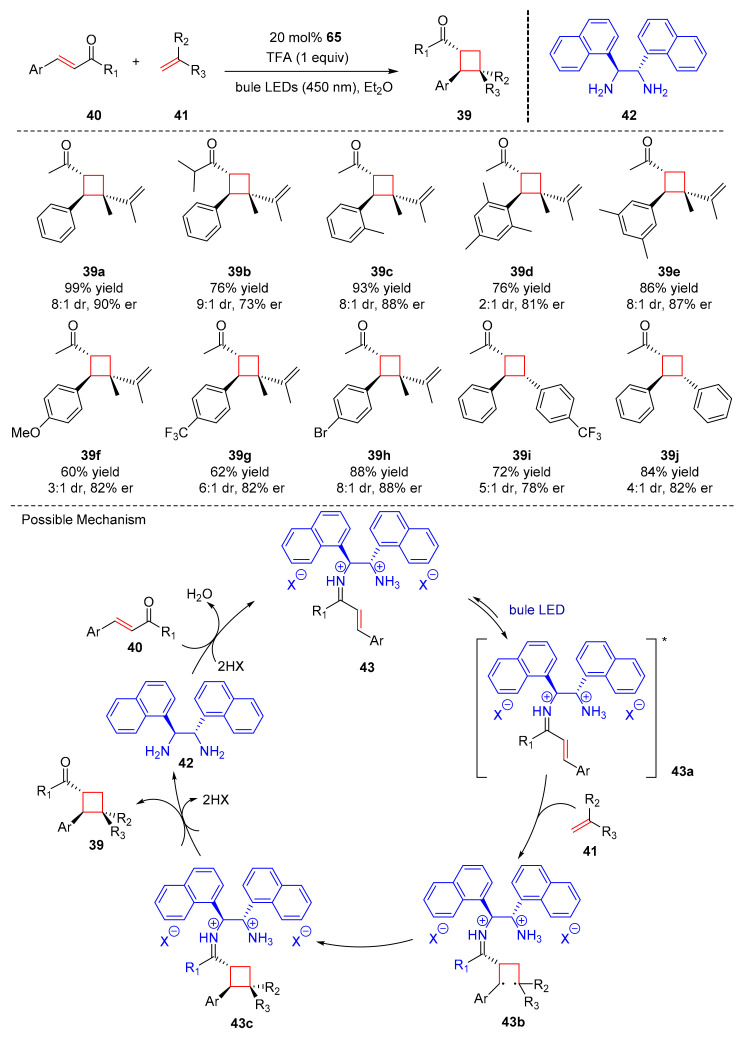
Enantioselective aminocatalytic [2+2] cycloaddition for cyclobutanes.

In 2020, Alemán et al. reported a novel enantioselective catalytic [2+2] cycloaddition to generate cyclobutanes (**39**) from α,β-unsaturated ketones (**40**) and olefins (**41**) via a diamine catalyst (**42**) upon the diffraction of blue LED light (456 nm) in good yields and enantioselectivity (43–99% yield, 72–91% er) (Scheme 10) [20]. TFA (trifluoroacetic acid) was used as an acidic promoter. The electron-donating and electron-withdrawing groups at *para*-position of aryl moiety could obtain similar result in enantioselectivity. Moreover, different olefins were also well tolerated. The proposed mechanism was conducted as follows. The first step was the cooperation of (**40**) with diamine catalyst (**42**) to form iminium ion intermediates (**43**), then the iminium ion intermediates transformed into (**43a**) upon the irradiation with blue LED light, which underwent photocycloaddition with (**41**) to form 1,4-biradical intermediates (**43b**). (**43b**) closed the ring and gave cyclobutyl iminium ions (**43c**) which could further release the desirable products (**39**) and finish a catalytic cycle.

**Scheme 11:**
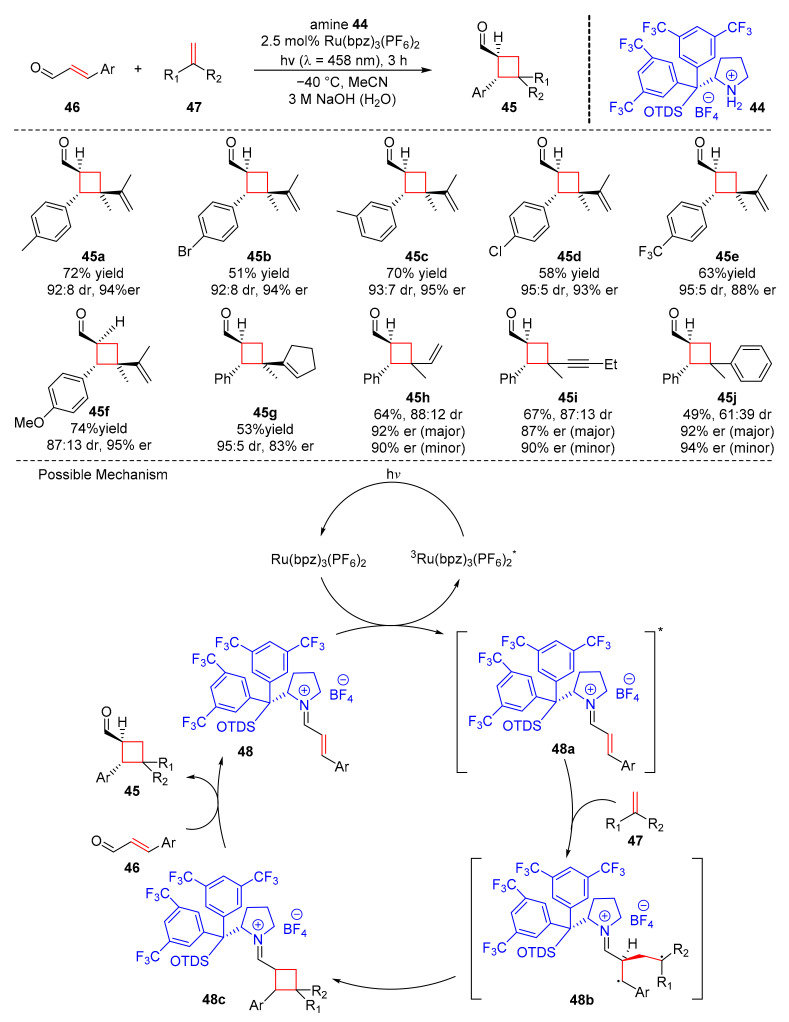
Enantioselective photocatalytic [2+2] cycloaddition of α,β-unsaturated aldehydes using chiral secondary amine and ruthenium catalyst.

Bach et al. found a co-catalytic system with ruthenium catalyst (Ru(bpz)_3_(PF_6_)_2_) and a chiral secondary amine catalyst (**44**) to synthesize cyclobutanecarbaldehydes (**45**) from α,β-unsaturated aldehydes (**46**) and olefins (**47**) upon the irradiation of visible light (λ = 458 nm) in moderate to good yields and enantioselectivity (49–74% yield, 83–96% er) (Scheme 11) [21]. A variety of substituents such as bromo, chloro, trifluoromethyl, methoxy, pinacolatoboryl, and acetoxy groups at the aryl moiety of α,β-unsaturated aldehydes are reliable substrates. Different olefins with a conjugated π-system were also tolerated. The proposed mechanism was conducted as follows. The α,β-unsaturated aldehydes (**46**) reacted with chiral amine catalyst to form eniminium ions (**48**), which could transform into the triplet intermediates (**48a**) through energy transfer from ruthenium catalyst at assistance of light. Subsequently, the addition of intermediates (**48a**) to olefins (**47**) generated 1,4-diradical intermediates (**48b**), which underwent intersystem crossing and hydrolysis to give the desired products (**45**) from (**48c**).

**Scheme 12:**
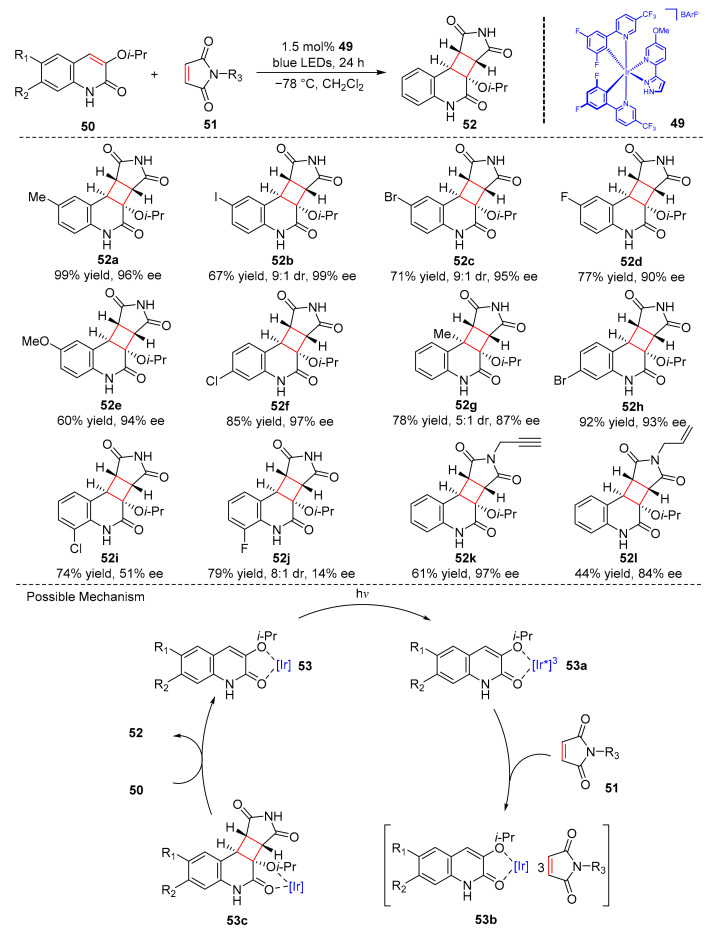
Enantioselective intermolecular [2+2] photocycloaddition of 3-hydroxyquinolones with maleimide.

Yoon et al. reported a highly enantioselective intermolecular [2+2] photocycloaddition reaction catalyzed by chiral hydrogen bond ion iridium photosensitizer (**49**). 3-Hydroxyquinolones (**50**) reacted with maleimide (**51**) to generate cycloaddition products (**52**) under the irradiation of blue LED light in excellent yields and enantioselectivity (up to 99% yield, up to 99% ee) (Scheme 12) [22]. The reaction has high enantioselectivity when the substitutions at the 6-position of 3-hydroxyquinolones are alkyl, halogen, and alkoxy groups, and the substituted 3-hydroxyquinolones at 5- and 7-positions also have good tolerance. However, the substitution at the 8-position has a great influence in enantioselectivity. Furthermore, the reaction is also applicable to alkyl, propyl, allyl, and carbamoyl substituted maleimide. In this reaction, the quinolone substrates (**50**) partially combined with the pyrazole of the iridium complex to afford complex (**53**), which was then transformed into an excited state (**53a**) under the irradiation of blue LED light. The excited state (**53a**) reacted with maleimide (**51**) by bimolecular energy transfer to obtain the complex (**53b**) and provided cycloaddition products (**52**) from (**53c**).

**Scheme 13:**
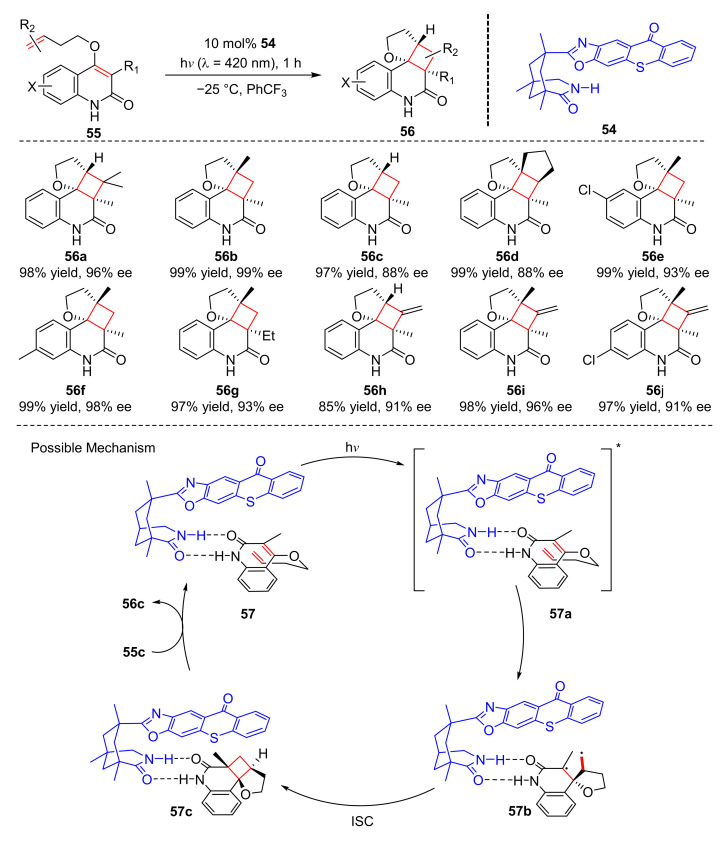
Enantioselective intramolecular cyclization of 3-alkylquinolones.

Recently, Bach et al. reported an enantioselective photoaddition reaction catalyzed by chiral thioxone (**54**). Under the irradiation of visible light (λ = 420 nm), intramolecular cyclization of 3-alkylquinolones (**55**) with 4-O-tethered alkenes or allenes occurred to form cycloaddition products (**56**) in good yields and enantioselectivity (72–99% yield, 81–99% ee) (Scheme 13) [23]. The benzo ring of quinolones substituted by methyl, chloro, cyano, methoxy, and fluoro groups has good tolerance. In the study of olefins, propylene diene and trifluoroolefins were also suitable for this reaction. The reaction mechanism shows that alkylquinolones (**55c**) could react with thioxanthraquinones (**54**) to deliver the complex (**57**) which gave the quinolone triplet (**57a**) by energy transfer, then the internal carbon atom of olefin was added to form 1,4-diradical (**57b**), which underwent intersystem crossing (ISC) to produce (**57c**) and further gave desired product (**56c**).

**Scheme 14:**
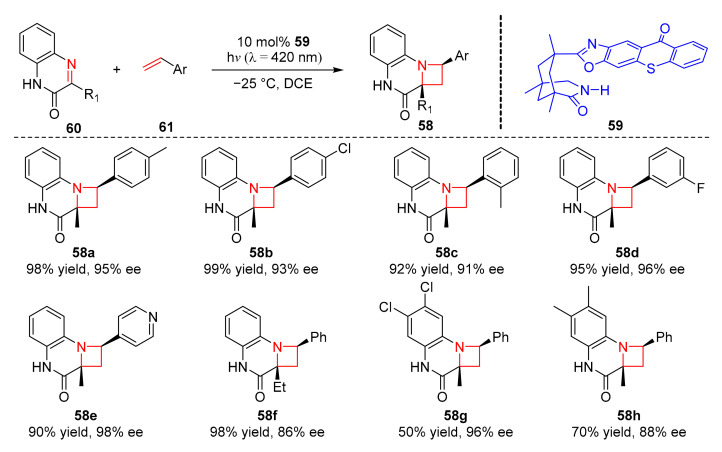
Intermolecular aza Paternó–Büchi reaction of 3-Substituted quinoxalin-2 (1H)-ones and olefins.

In 2020, Bach et al. reported a photocyclic addition reaction in which heterocyclic compounds (**58**) could be synthesized, using thioxanthone (**59**) as a chiral catalyst. Under the irradiation of visible light (λ = 420 nm), 3-substituted quinoxalin-2 (1H)-ones (**60**) and olefins (**61**), could occur an intermolecular aza Paternó–Büchi reaction in good yields and enantioselectivity (50–99% yield, 86–98% ee) (Scheme 14) [24]. The *para*-position of olefin aromatic ring could be substituted by some groups such as methyl, tert-butyl, and halogen substituents. Ethyl and trifluorocarbons at the C3 of quinoxalinones were also well tolerated. The reaction mechanism is similar to the previously mentioned mechanism (Scheme 13).

**Scheme 15:**
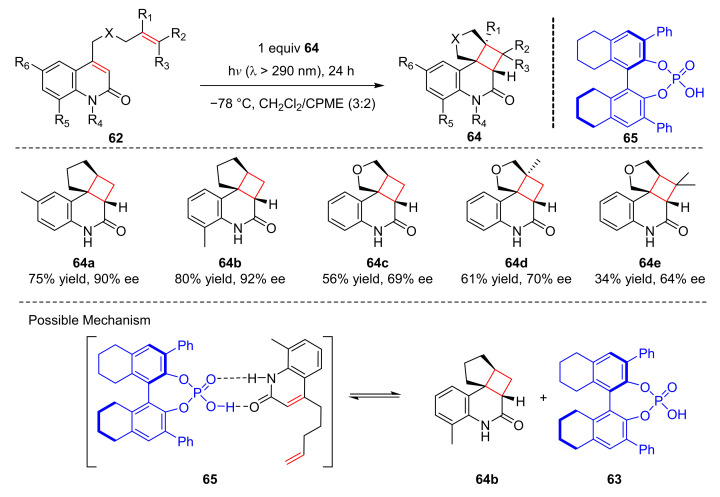
Enantioselective intramolecular [2+2] photocycloaddition of 4-bishomoally-2-quinolones.

In 2020, Takagi et al. reported an enantioselective intramolecular [2+2] photocycloaddition of 4-bishomoally-2-quinolones (**62**). When phosphoric acid (**63**) was used as a photocatalyst, cycloaddition products (**64**) were obtained with good yields and enantioselectivity (up to 88% yield, up to 92% ee) under the irradiation of visible light (λ > 290 nm) (Scheme 15) [25]. Methyl groups at the 6- and 8-positions of the substrates were well tolerated, while oxygen atoms could reduce the enantioselectivity of the products. The reaction occurred from a complex (**65**) formed by substrate (**62b**) and phosphoric acid (**63**) through dual hydrogen bonding, then the olefin moiety on the complex reacted with the enone moiety to form cycloadduct (**64b**) via photocycloaddition.

**Scheme 16:**
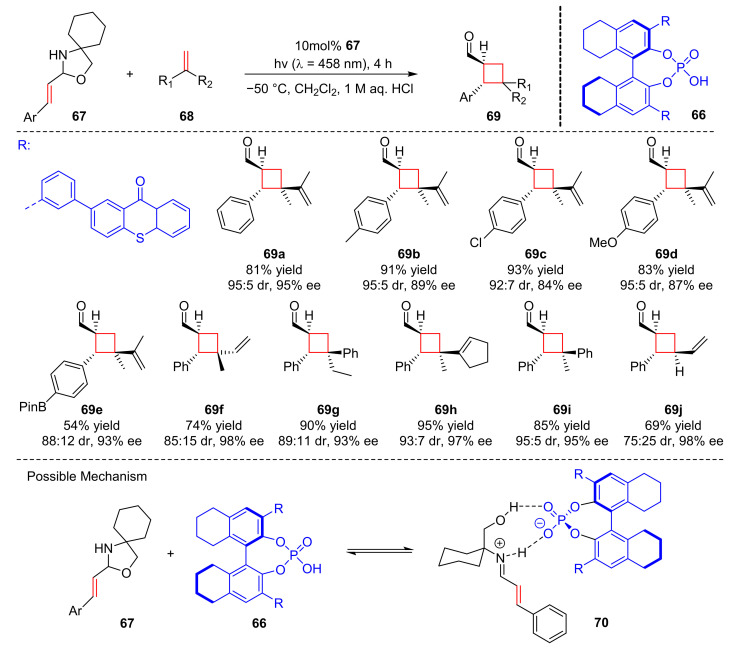
Enantioselective [2+2] photocycloaddition of N,O-acetals and olefins.

Very recently, Bach et al. reported another enantioselective [2+2] photocycloaddition catalyzed by chiral phosphoric acid (**66**). Under the irradiation of visible light (λ = 459 nm), the reaction could combine N,O-acetals (**67**) with olefins (**68**) to form cyclobutanecarbaldehydes (**69**) in good yields and enantioselectivity (54–96% yield, 84–98% ee) (Scheme 16) [26]. The *para*-substituents on the phenyl ring, such as methyl, bromine, chloro, methoxy, and borate group, were well tolerated. In different olefins, styrenes, 1,3-enynes and 1,3-dienes could produce products with good enantioselectivity. The author’s study shows that the reaction could be carried out due to the formation of the complex intermediates (**70**), which were combined by the substrates (**67**) and the catalyst (**66**).

**Scheme 17:**
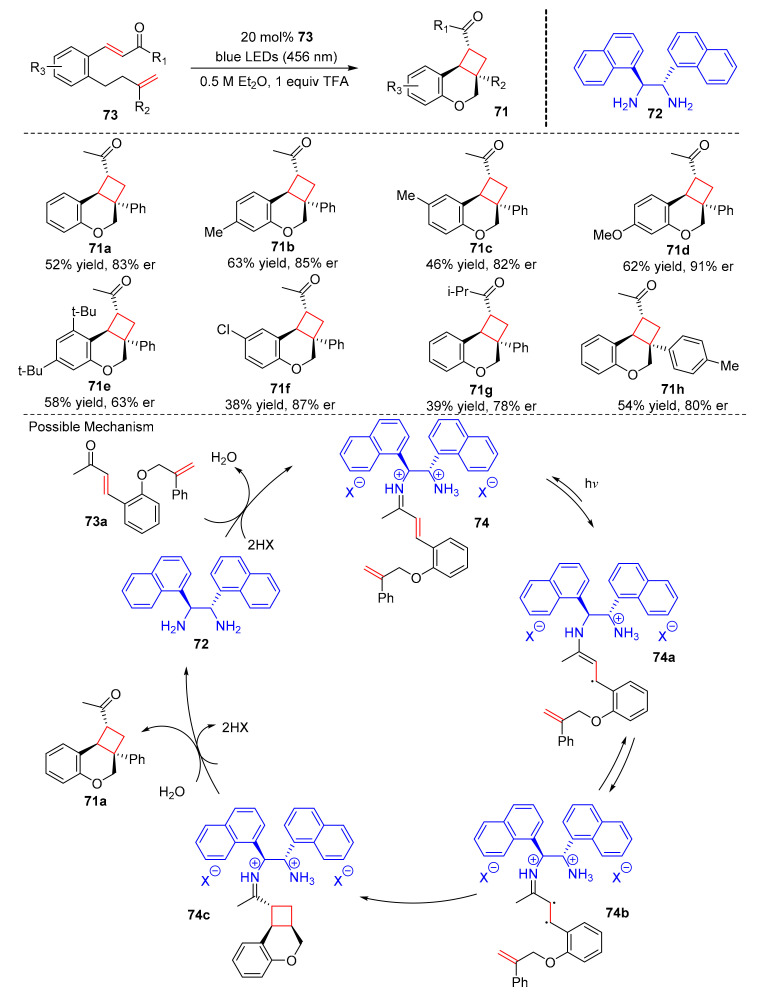
Intramolecular [2+2] photocycloaddition for the synthesis of polycyclic ethers containing cyclobutane.

In 2021, Alemán et al. reported an intramolecular [2+2] photocycloaddition for the synthesis of polycyclic ethers (**71**) containing cyclobutane (Scheme 17). The reaction was catalyzed by amines (**72**) which could easily convert into iminium ions. Under the irradiation of blue light (λ = 459 nm), the modified salicylaldehydes (**73**) could be successfully obtained in good yields and enantioselectivity (38–63% yield, 65–91% er) [27]. The enantioselectivity of the products could be improved if there are strong electron-donating groups at the aryl group of the salicylaldehyde core. Different aryl groups in the styrene chain, such as methyl and fluoryl, could facilitate a smooth reaction. The proposed mechanism was conducted as follows. First, the substrates (**73a**) combined with the catalysts (**72**) to furnish iminium ion intermediates (**74**). The excited complex (**74a**) was formed by SET under the irradiation of blue light LED, then the excited complex (**74a**) could transform into biradical intermediates (**74b**), which underwent [2+2] photocycloaddition to give the cyclobutyl iminium ions (**74c**). Finally, the cyclobutyl iminium ions (**74c**) produced the desired product, (**71a**).

**Scheme 18:**
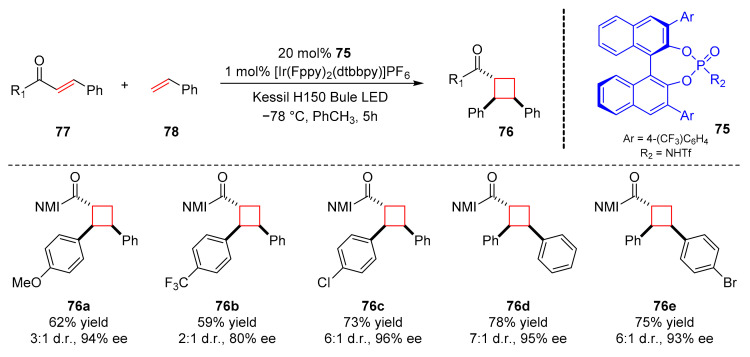
Asymmetric [2+2] photocycloaddition from α,β-unsaturated carbonyl compounds and alkenes.

In 2021, Yoon et al. used a chiral Brønsted acid (**75**) to obtain cyclobutane products (**76**) by asymmetric [2+2] photocycloaddition from α,β-unsaturated carbonyl compounds (**77**) and alkenes (**78**) in good yields and enantioselectivity (up to 85% yield, up to 99% ee) (Scheme 18) [28]. The reaction shows wide applicability, and some medicinally relevant motifs were also synthesized. It is worth noting that cyclobutane products have trans–cis stereochemical properties which could be complementary to other enantioselective [2+2] photocycloadditions.

### 2.3. Enantioselective Formation of 5-Membered Ring by Visible Light Catalysis

**Scheme 19:**
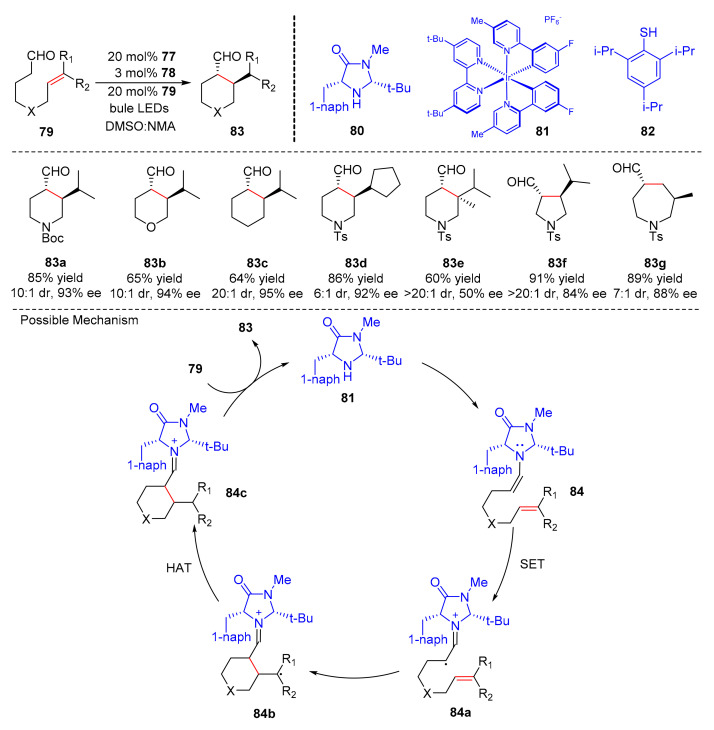
Intramolecular α-alkylation of aldehydes via a co-catalytic system.

In nature, 5-Membered ring compounds exist widely, and some five-membered heterocyclic compounds, such as furan, pyrrole, and thiophene, are widely used in organic synthesis and have a variety of physiological activities as drugs. In 2017, MacMillan et al. reported an intramolecular α-alkylation of aldehydes (**79**) via a co-catalytic system (amine catalyst (**80**), iridium photocatalyst (**81**) and HAT catalyst (**82**)) to obtain five-membered, six-membered, or seven-membered cyclic aldehydes (**83**) under the irradiation of blue LED light in good yields and enantioselectivity (up to 91% yield, up to 95% ee) (Scheme 19) [29]. This reaction could be used to prepare a variety of heterocyclic compounds containing nitrogen atoms and synthesize tetrahydropyran. In the scope of alkenes, trisubstituted and 1,2-disubstituted olefins were well tolerated. The proposed mechanism was conducted as follows. The substrates (**79**) combined with amine catalyst (**80**) to afford enamines (**84**). At the same time, under the irradiation of visible light, enamines (**84**) formed electrophilic radical (**84a**) through SET initiated by iridium photocatalyst, which was added to olefins to produce nucleophilic radical (**84b**). Nucleophilic radical (**84b**) underwent HAT to generate iminium ions (**84c**); finally desired products (**83**) were obtained by releasing amine catalyst (**80**) from iminium ions (**84c**).

**Scheme 20:**
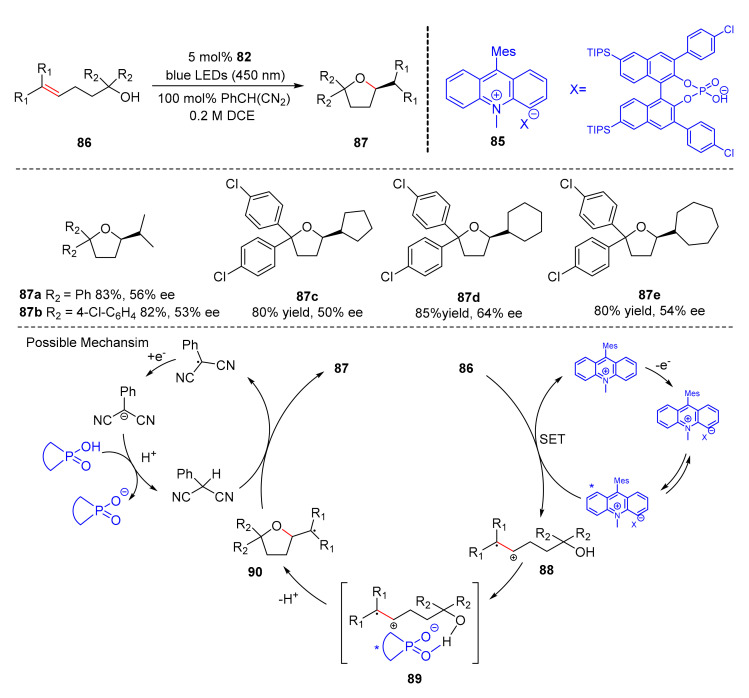
Enantioselective anti-Markovnikov hydroetherification of alkenols.

In 2017, Luo et al. reported a chiral ion-pair photoredox organocatalyst (**85**) which was used for enantioselective anti-Markovnikov hydroetherification of alkenols (**86**) to synthesize five-membered oxygen-containing heterocyclic adducts (**87**) under the irradiation of bule LED light (λ = 450 nm) in good yields and enantioselectivity (50–90% yield, up to 64% ee). The chiral ion-pair is composed of chiral BINOL-based sodium phosphate and 9-mesityl-10-methylacridinium tetrafluoroborate (Scheme 20) [30]. The aryl substitutions of hydroxyl α-position were well tolerated. Studies have shown that the reaction begins with the chiral ion-pair-catalyzed SET step, which converts the substrates (**86**) into radical intermediates (**88**), radical intermediates (**88**) combine with chiral phosphate anion to form complex (**89**), and complex (**89**) undergo cyclization to yield cyclic adducts (**90**), which through chiral phosphate anion mediated hydrogen transfer to give desired products (**87**).

**Scheme 21:**
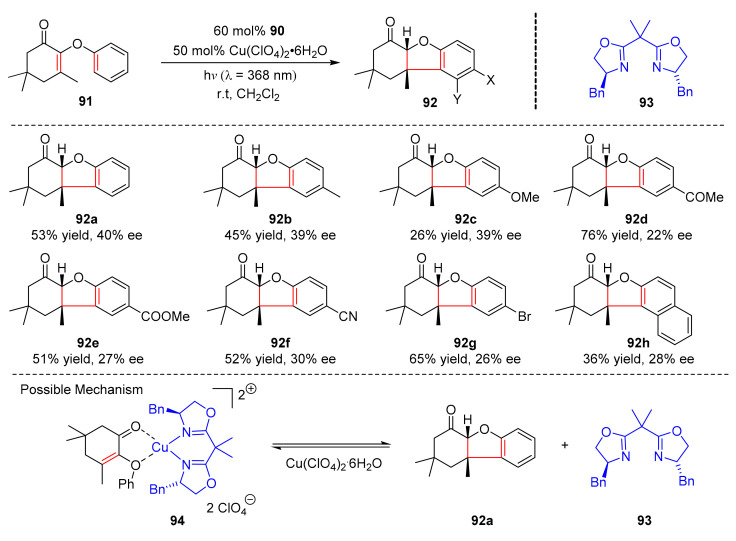
Enantioselective photocyclization reaction of 2-aryloxy-cyclohex-2-enones.

In 2017, Bach et al. reported an enantioselective photocyclization reaction which converted 2-aryloxy-cyclohex-2-enones (**91**) to cis-2,3,4a,9b-tetrahydro-1H-dibenzofuran-4-ones (**92**) in moderate yields and enantioselectivity (26–76% yield, up to 60% ee) (Scheme 21) [31]. In the presence of Cu(ClO_4_)_2_·6H_2_O and bisoxazoline ligand (**93**), the reaction could be carried out under the irradiation of visible light (λ = 368 nm), or under the irradiation of visible light (λ = 418 nm) with the addition of 50 mol% of thioxanthone. The electron-donating groups on the aryl *para*-position have no effect, while the electron-withdrawing groups lead to the decrease in enantioselectivity. Studies have shown that the substrate (**91a**) could form the complex (**94**) with chiral copper-bisoxazoline complex so that the β-carbon atoms of ketene could be attacked to generate cyclic adducts (**92a**).

**Scheme 22:**
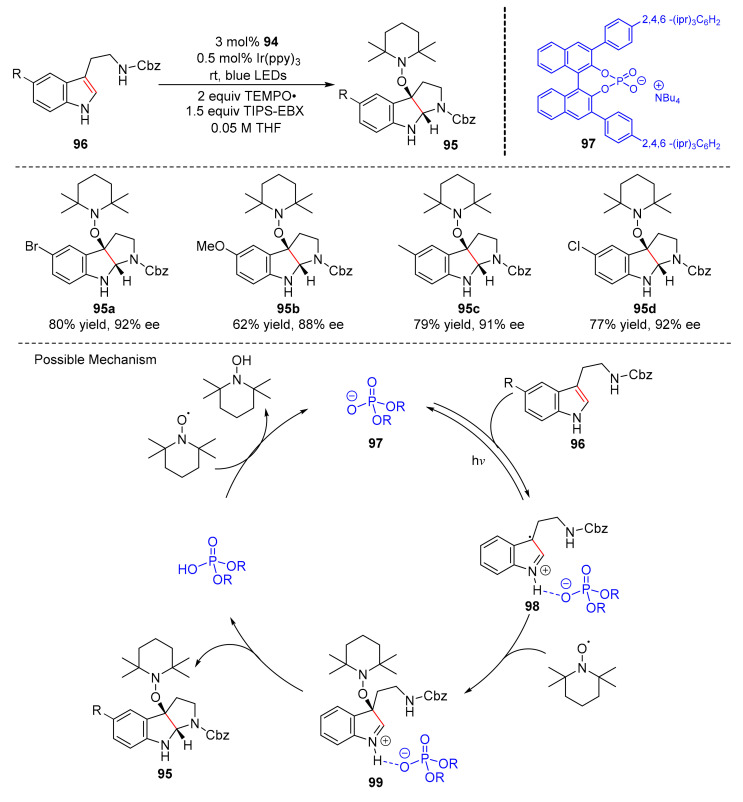
Enantioselective synthesis of pyrroloindolines.

In 2018, Knowles et al. reported a photocatalytic reaction to synthesize pyrroloindolines (**95**) from tryptamine substrates (**96**) under the irradiation of blue LED light in good yields and enantioselectivity (59–81% yield, 87–92% ee) (Scheme 22) [32]. Ir(ppy)_3_ and 8H-TRIP BINOL phosphate (**97**) were used as catalysts. Some substituents on the indole core were well tolerated, such as Br-, Cl-, Methoxy-, and alkyl-substituents. Moreover, the reaction could also be applied to the synthesis of alkaloid natural products. The proposed mechanism was conducted as follows. Chiral phosphates could first form hydrogen-bonded adducts (**98**) with substrates (**96**). Under the irradiation of visible light, electron transfer occurred and reacted with stable nitroxyl TEMPO· to produce closed-shell intermediates (**99**); iminium ions underwent nucleophilic addition with pendant amine to obtain alkoxyaminesubstituted pyrroloindoline products (**95**).

**Scheme 23:**
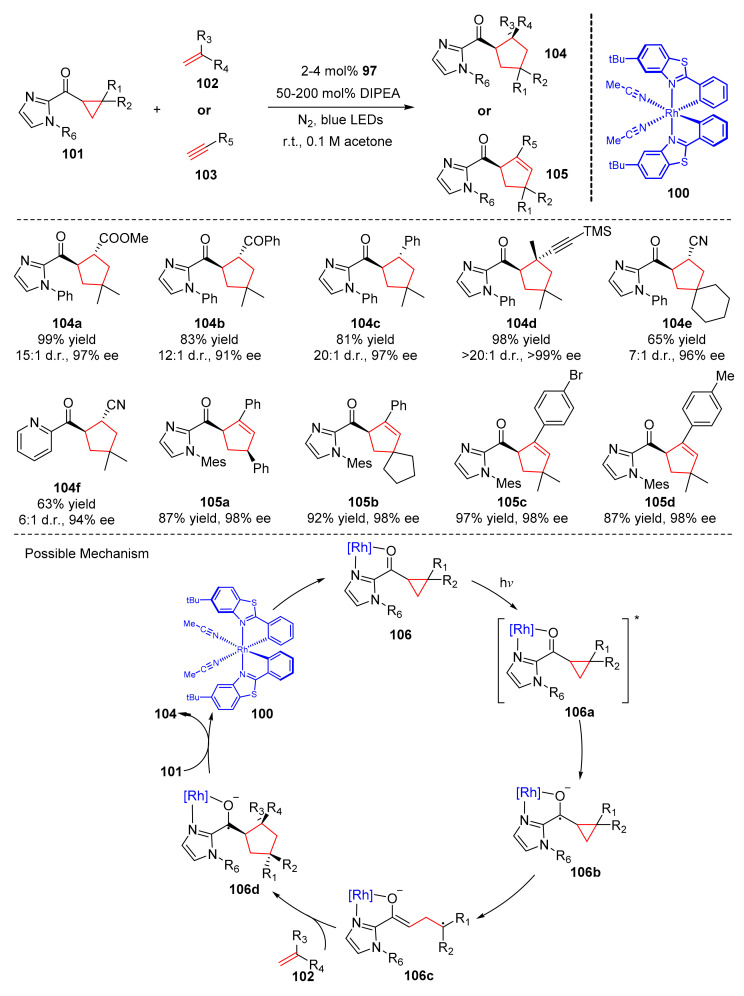
[3+2] photocycloadditions of cyclopropanes with alkenes or alkynes.

In 2018, Meggers et al. reported a [3+2] photocycloaddition catalyzed by chiral-at-metal rhodium complex (**100**). Under the irradiation of bule LED light, cyclopropanes (**101**) reacted with alkenes (**102**) or alkynes (**103**) to deliver chiral cyclopentanes (**104**) or cyclopentenes (**105**) in good yields and enantioselectivity (63–99% yields, up to >99% ee) (Scheme 23) [33]. Alkenes have a wide range of applicability, and the olefins substituted by Michael acceptors, styrenes, enynes, and aromatic rings were well tolerated; pyridine could also be used as a substituent group to participate in the reaction. In the scope of alkynes, various aryl substituted alkynes were well tolerated. The proposed mechanism was conducted as follows. Bidentate coordination occurred between cyclopropane substrates (**101**) and rhodium complex RhS (**100**) to generate intermediates (**106**), which were excited to intermediates (**106a**) under the irradiation of visible light. Intermediates (**106a**) as a strong oxidant were reduced to intermediates (**106b**) by tertiary amine. Intermediates (**106b**) were converted into radical intermediates (**106c**), which were added to alkenes (**102**) to generate ketyl radical (**106d**). Then ketyl radical (**106d**) released cycloaddition products (**105**) to complete catalytic cycle.

**Scheme 24:**
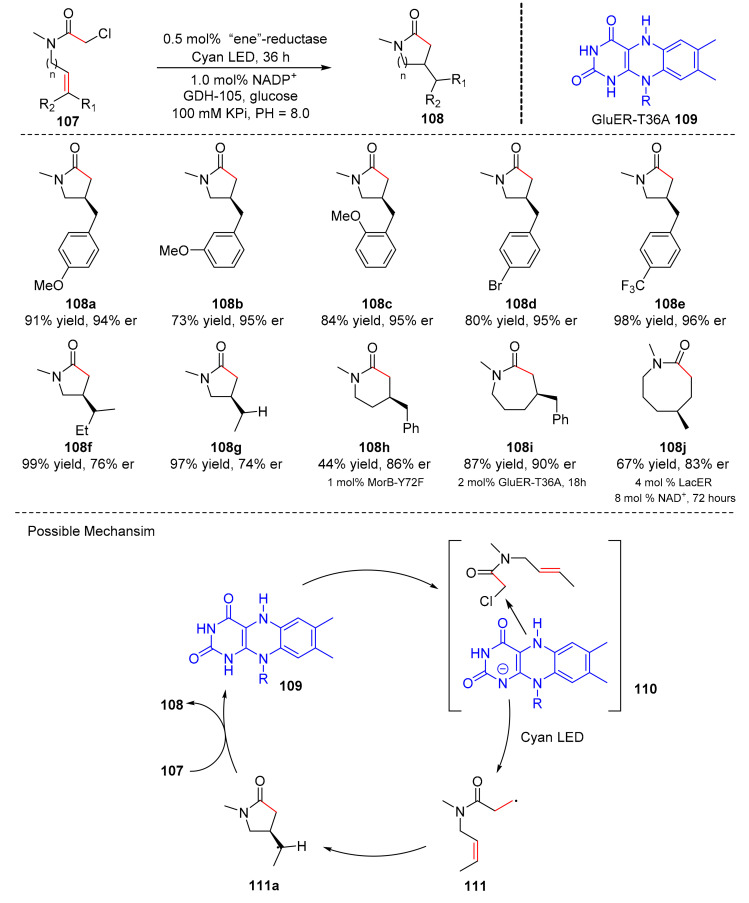
Enantioselective photocyclization of chloroacetamides.

In 2019, Hyster et al. reported a photoexcitation catalyzed by flavin-dependent “ene”-reductase. The methodology could convert chloroacetamides (**107**) to five-, six-, seven-, and eight-membered lactams (**108**) under the irradiation of 50 W cyan light (λ = 497 nm) in good yields and enantioselectivity (up to 99% yield, up to >99% er), GluER-T36A (**109**) is used as the main chiral catalyst (Scheme 24) [34]. Aromatic substituted alkenes could participate in this reaction smoothly, and a variety of alkyl substituents on the olefin were well tolerated. The proposed mechanism was conducted as follows. Substrates (**108**) could combine with catalyst (**109**) to yield complex (**110**), which underwent electron transfer to obtain radical intermediates (**111**). Intermediates (**111**) formed exocyclic radical (**111a**) via cyclization, which gave desired products (**108**) through hydrogen atom transfer.

**Scheme 25:**
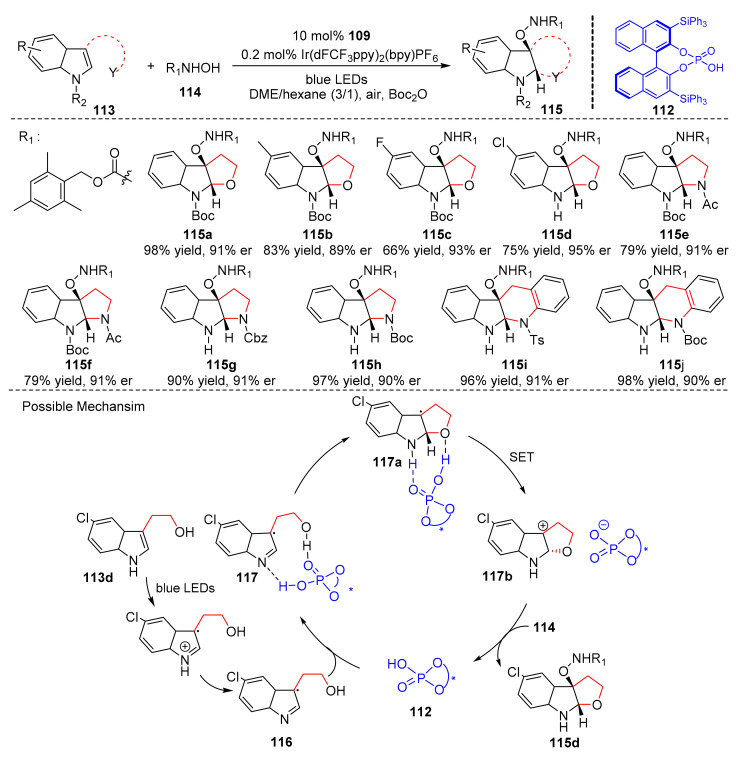
Asymmetric dearomatization of substituted indolines and N-hydroxycarbamates.

In 2019, You et al. reported an asymmetric dearomatization catalyzed by an iridium photocatalyst and a chiral phosphoric acid (**112**). Under the irradiation of blue LED light, substituted indolines (**113**) could react with *N*-hydroxycarbamates (**114**) to form cyclic adducts (**115**) in good yields and enantioselectivity (up to 98% yield, up to 96% er) (Scheme 25) [35]. Many kinds of substituted furoindolines and pyrroloindolines were produced by this reaction with high enantioselectivity. In addition, substituted indolo[2,3-*b*]quinolines could also be constructed. The proposed mechanism was conducted as follows. In the presence of an iridium photocatalyst, substrate (**113d**) went through first SET oxidation to form radical cation intermediate (**116**) under the irradiation of visible light, which cooperated with chiral phosphate anion (**112**) to generate complex (**117**); then, complex (**117**) converted to complex (**117a**) through CPA-mediated cyclization. Complex (**117a**) underwent second SET oxidation to produce tertiary pyrroloindoline carbocation intermediate (**117b**). Finally, tertiary pyrroloindoline carbocation intermediate (**117b**) reacted with *N*-hydroxycarbamate (**114**) to obtain desired product (**115d**).

**Scheme 26:**
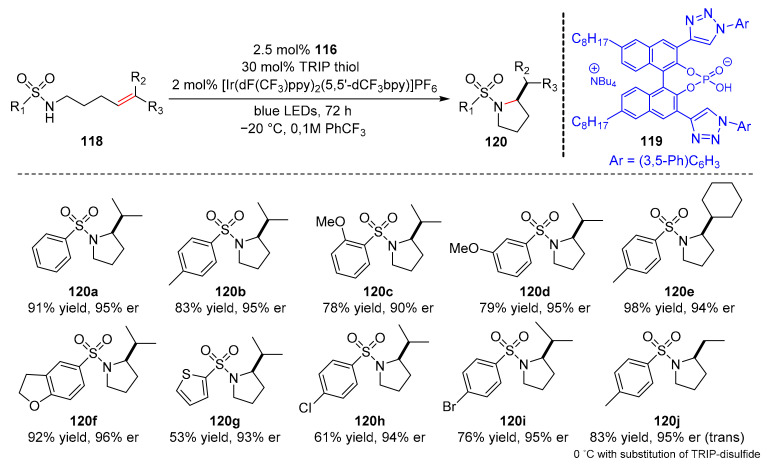
Enantioselective intramolecular hydroamination of alkenes with sulfonamides.

In 2020, Knowles et al. reported a kind of enantioselective intramolecular hydroamination of alkenes with sulfonamides (**118**) catalyzed by an iridium photocatalyst and a chiral phosphate (**119**). Pyrrolidines (**120**) were successfully obtained under the irradiation of blue LED light in good yields and enantioselectivity (up to 98% yield, up to 98% er) (Scheme 26) [36]. In the scope of sulfonamide moieties, the substituents at *para*- and *meta*-position of the sulfonamide arenes could provide products with high er; the reaction could also be suitable for benzofuran, thiophene, and thiazole heterocycles. Benzyl substitution, phenethyl chain, sulfamate ester, and sulfamide substrates were well tolerated. In addition, some complex sulfonamide substrates could also participate in this reaction. For the scope of alkenes, cyclohexyl-substituted and cyclobutyl-substituted substrates showed better enantioselectivity.

**Scheme 27:**
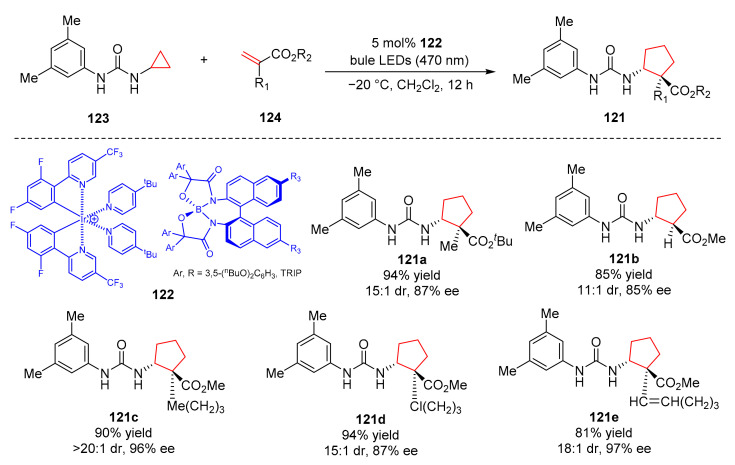
Asymmetric synthesis of 5-membered alicyclic α-quaternary β-amino acids via chiral iridiumborate-catalysed [3+2] photocycloaddition.

In 2021, Ooi et al. reported an asymmetric synthesis of 5-membered alicyclicα-quaternary β-amino acids (**121**) [37], which relies on the chiral iridiumborate (**122**)-catalyzed [3+2] photocycloaddition of cyclopropylurea (**123**) with α-substituted acrylates (**124**). Under the irradiation of bule light (λ = 470 nm), the reaction showed good yields and enantioselectivity (up to 97% yield, up to 97% ee) (Scheme 27). Various functional groups such as terminal olefins, chlorine, ethers, and esters on the α-position of acrylates were well tolerated.

### 2.4. Enantioselective Formation of 6-Membered Ring by Visible Light Catalysis

**Scheme 28:**
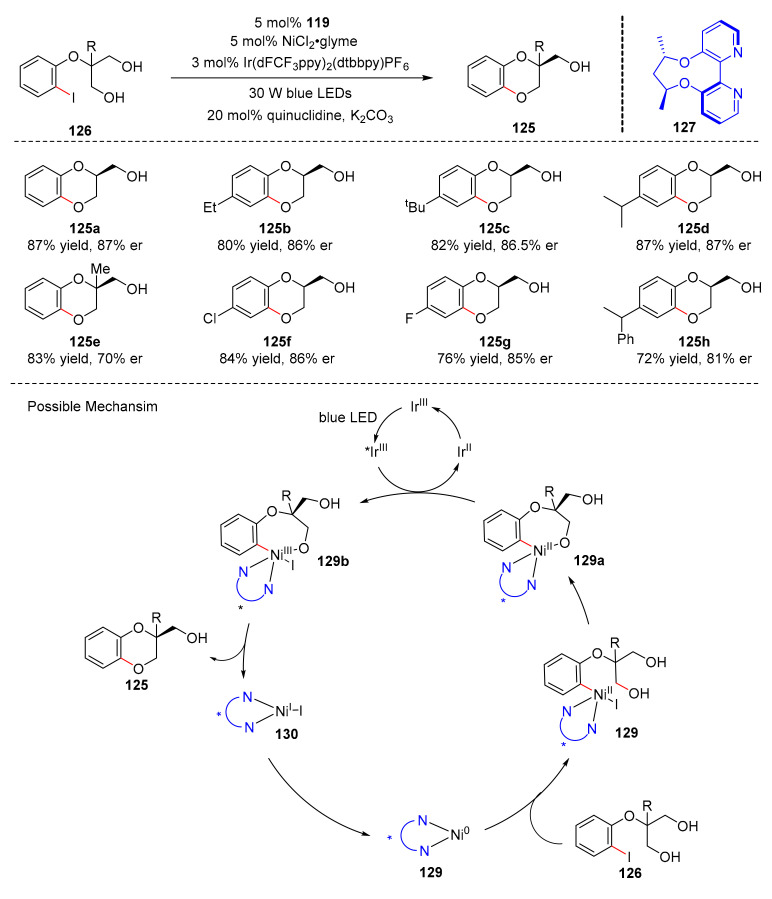
Desymmetric C-O coupling reaction for the enantioselective synthesis of 1,4-benzodioxanes.

Six-membered ring compounds are also a kind of very important organic compounds, especially six-membered heterocyclic compounds which play a significant role in the field of drug synthesis. In 2018, Xiao et al. reported a dual photoredox and nickel catalyzed desymmetric C–O coupling reaction to synthesize enantioselective 1,4-benzodioxanes (**125**) from 2-(2-iodophenoxy) propane-1,3-diol analogues (**126**) under the irradiation of blue LED light in good yields and enantioselectivity (up to 87% yield, up to 88% er) (Scheme 28) [38]. NiCl_2_·glyme and Ir(dFCF_3_ppy)_2_(dtbbpy)PF_6_ were selected as catalysts, and axially chiral 2,2′-bipyridine (**127**) was used as ligand. Halidecontained substrates were well tolerated, this reaction could also be used to synthesize 1,4-benzodioxane containing oxa-quaternary stereocenters. The mechanism proposed by the authors was conducted as follows. Substrates (**126**) could undergo oxidative addition with chiral Ni(0) complex (**128**) to form Ni(II) aryl complex (**129**), which could give Ni(II) aryl alkoxides (**129a**) under the action of chiral 2,2′-bipyridine ligand. Ni(II) aryl alkoxides (**129a**) formed critical Ni(III) aryl alkoxides (**129b**) through iridium(III) photocatalyst mediated SET process. Finally, critical Ni(III) aryl alkoxides (**129b**) underwent reductive elimination to release desired products (**125**) and chiral Ni(I) complex (**130**).

**Scheme 29:**
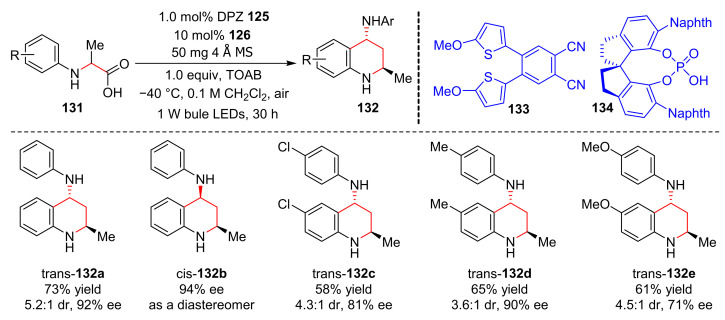
Photoredox dual-catalysis for synthesis of 4-amino-2methyl THQs.

In 2018, Jiang et al. reported a photoredox dual-catalysis, which converted *N*-aryl α-amino acids (**131**) into chiral 4-amino-2methyl THQs (**132**) under the irradiation of blue LED light in good yields and enantioselectivity (58–73% yields, 71–92% ee) for the first time (Scheme 29) [39]. Dicyanopyrazine-derived chromophore (DPZ) (**133**) and a chiral phosphate (**134**) were utilized as co-catalysts. In the study of the reaction range, the benzene ring of substrates substituted by some groups, such as Br-, Me-, and MeO-, were well tolerated.

**Scheme 30:**
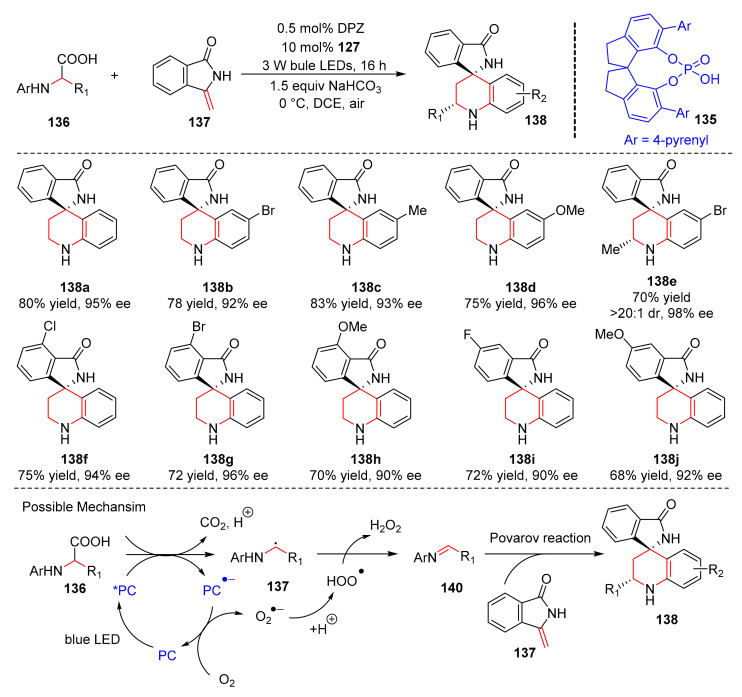
Cooperative photoredox and chiral Brønsted acid catalysis for synthesis of chiral isoindolin-1-ones.

Later, Jiang et al. reported a kind of cooperative photoredox and chiral Brønsted acid catalysis. Under the irradiation of bule LED light, *N*-aryl amino acids (**136**) react with 3-methyleneisoindolin-1-ones (**137**) to form chiral isoindolin-1-ones (**138**) in good yields and enantioselectivity (up to 83% yield, up to 98% ee) in the presence of a chiral phosphoric acid (**135**) and DPZ as catalysts (Scheme 30) [40]. A variety of substituents (Br-, Me-, MeO-, etc.) on the aromatic rings of THQ moieties could obtain products with high enantioselectivity. In the scope of methylenephthalimidines, substituents on the benzene ring, such as Cl-, Br-, F-, and MeO-, were well tolerated. The proposed mechanism was conducted as follows. *N*-aryl amino acids (**136**), oxidized by a photoredox catalyst, underwent proton transfer and decarboxylation to form a-aminoalkyl radical (**139**); α-aminoalkyl radical (**139**) transferred an electron to HO_2_· to furnish N-aryl imines (**140**), which reacted with 3-methyleneisoindolin-1-ones (**137**) to produce chiral isoindolin-1-ones (**138**) via chiral Brønsted acid-mediated Povarov reaction.

**Scheme 31:**
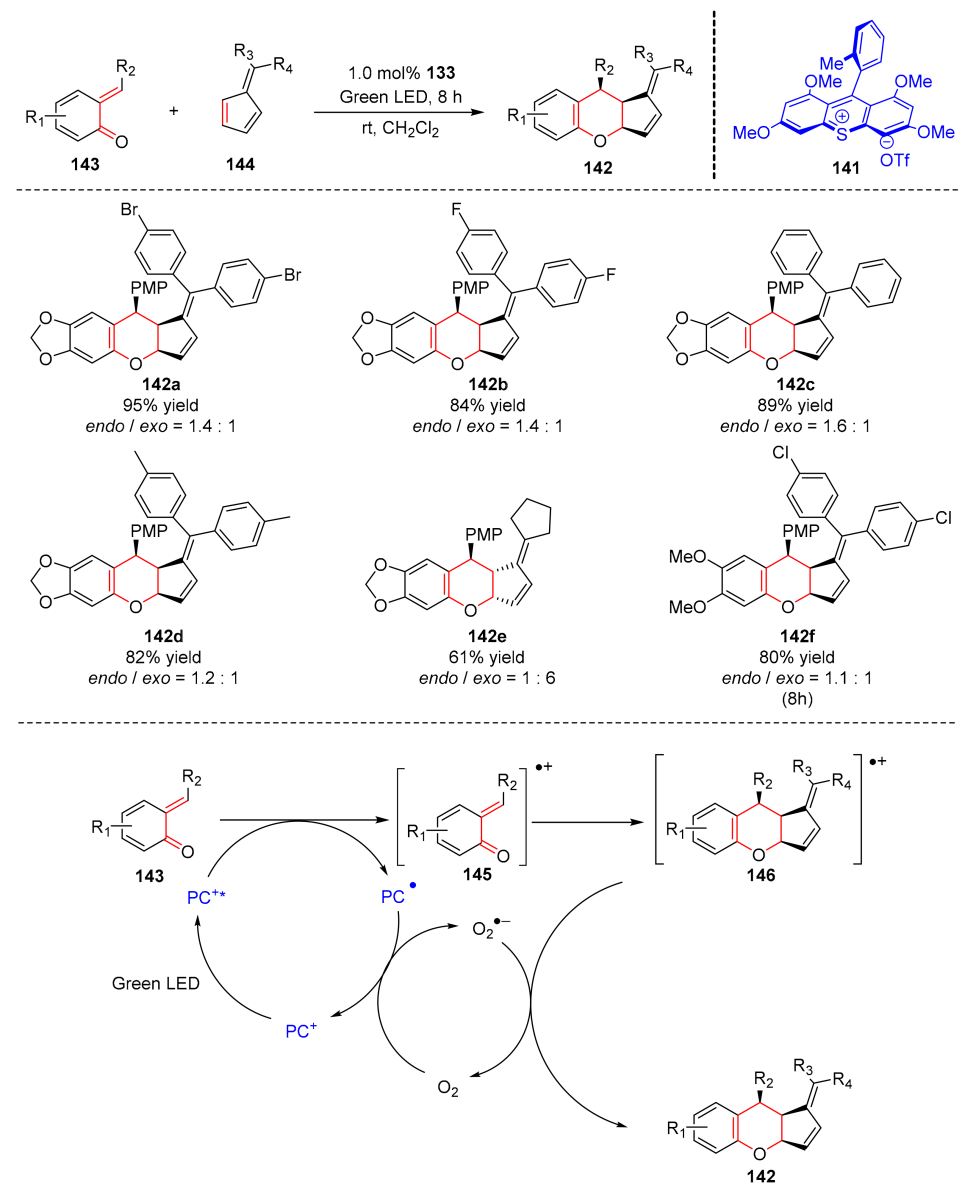
Organic photoredox catalyzed [4+2] cycloaddition of *ortho*-quinone methides and fulvenes.

In 2020, Honda et al. reported a thioxanthylium (**141**) organic photoredox catalyzed [4+2] cycloaddition to form tetrahydrocyclopenta[b]chromenes (**142**) from *ortho*-quinone methides (**143**) and fulvenes (**144**) under the irradiation of green light in good yields and enantioselectivity (up to 95% yield, up to 1.6:1 ee) (Scheme 31) [41]. In the study of the range of fulvenes, diarylfulvenes with halogen functionalities and electron-donating groups could obtain products smoothly. Fulvenes containing aliphatic groups were nice candidates. In addition, *ortho*-quinone methide with dimethoxy groups could also result in ideal products. The mechanism proposed by the authors was conducted as follows. Under the irradiation of green light, the excited photoredox catalyst oxidized substrates (**143**) to form radical cations (**145**). At the same time, the photoredox catalyst converted O_2_ to O_2_·^−^ through single electron transfer, radical cations (**145**) underwent [4+2] cycloaddition with fulvenes (**144**) to obtain radical cations (**146**). Finally, radical cations (**146**) produced cycloadducts (**142**) under the reaction of superoxide radical (O_2_·^−^).

**Scheme 32:**
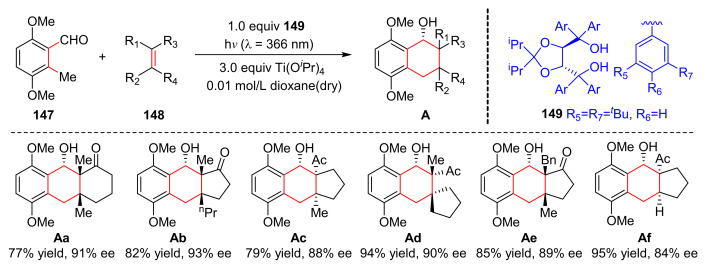
Construction of polycyclic structures from substituted 2-methylbenzaldehydes and dienophiles.

In 2021, Gao et al. reported a method for the construction of polycyclic structures (**A**) from substituted 2-methylbenzaldehydes (**147**) and dienophiles (**148**) via a chiral titanium (**149**)-mediated enantioselective photoenolization/Diels–Alder reaction [42]. The reaction has good yields and enantioselectivity (up to 98% yield, up to 99% ee) (Scheme 32), and could be used to synthesize a variety of complex natural products and drugs.

**Scheme 33:**
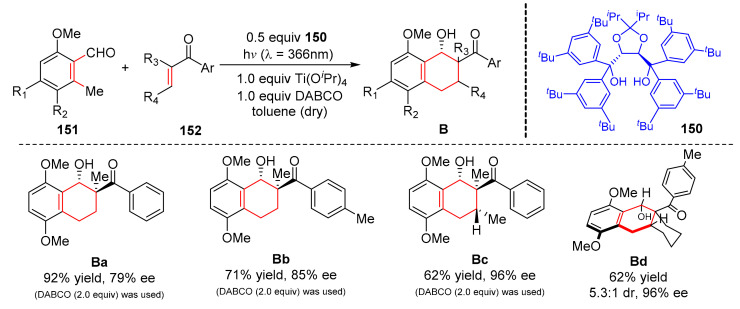
TADDOL-type ligand-mediated exo-selective and enantioselective photoenolization/Diels–Alder reaction.

Not long before, another chiral TADDOL-type ligand (**150**) for exo-selective and enantioselective photoenolization/Diels–Alder reaction was found by Gao et al. [43]. Under the irradiation of visible light (λ = 366 nm), electron-rich 2-methylbenzaldehydes (**151**) reacted with dienophiles containing a benzoyl group at its α position (**152**) to form a variety of D-A addition products (**B**) in good yields and enantioselectivity (up to 92% yield, up to 99% ee) (Scheme 33). The process of the reaction depended on the generation of the structure of the dienophiles and the chiral ligands, and the chiral dinuclear Ti-TADDOLate species provided an excellent enantioselective environment for [4+2] cycloaddition.

### 2.5. Enantioselective Formation of 7-Membered Ring by Visible Light Catalysis

**Scheme 34:**
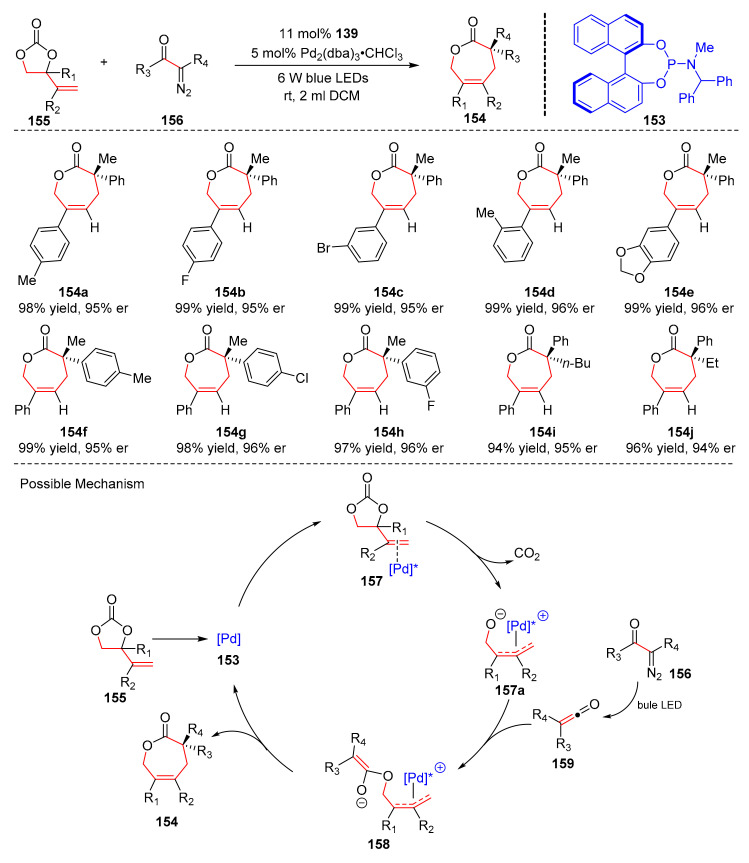
Enantioselective [5+2] cycloaddition of vinylethylene carbonates and α-diazoketones.

In 2019, Xiao et al. found an enantioselective [5+2] cycloaddition, merging a chiral ligand (**153**) with a Pd catalyst to synthesize chiral 7-membered lactones (**154**) from vinylethylene carbonates (**155**) and α-diazoketones (**156**) upon the diffraction of blue LED light in excellent yields and enantioselectivity (up to 99% yield, up to 96% er) (Scheme 34) [44]. Varied groups at aryl moiety of vinylethylene carbonates could obtain high enantioselective products, substituted α-diazoketones were well tolerated in this reaction. The proposed mechanism was conducted as follows. Vinylethylene carbonates (**155**) could react with Pd catalyst to generate complex (**157**), then complex (**157**) could transform into Pd-containing π-allyl dipolar intermediates (**157a**), which underwent the nucleophilic addition to give zwitterionic intermediates (**158**) with ketene species (**159**) from the Wolff rearrangement of α-diazoketones (**156**). Finally, zwitterionic intermediates (**158**) transformed into 7-membered lactones (**154**) via an intramolecular asymmetric allylic alkylation (AAA) reaction.

**Scheme 35:**
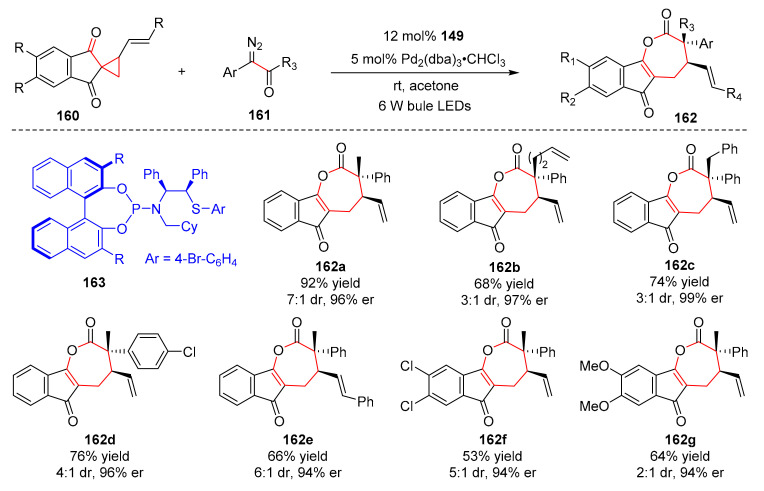
Asymmetric [5+2] dipolar cycloadditions of vinylcyclopropanes and α-diazoketones.

In 2020, Xiao et al. found a new asymmetric [5+2] dipolar cycloadditions of vinylcyclopropanes (**160**) and α-diazoketones (**161**) to synthesize 7-membered lactones (**162**) upon the diffraction of blue LED light in good yields and enantioselectivity (52–92% yields, up to 99% er) (Scheme 35) [45]. Pd_2_(dba)_3_·CHCl_3_ and a chiral ligand (**163**) acted as catalysts. Notably, substitutions the alkyl chain of lactones by some functional groups, such as alkenyl, alkynyl, and OTBS, were well tolerated in this reaction.

### 2.6. Enantioselective Formation of Macroring by Visible Light Catalysis

**Scheme 36:**
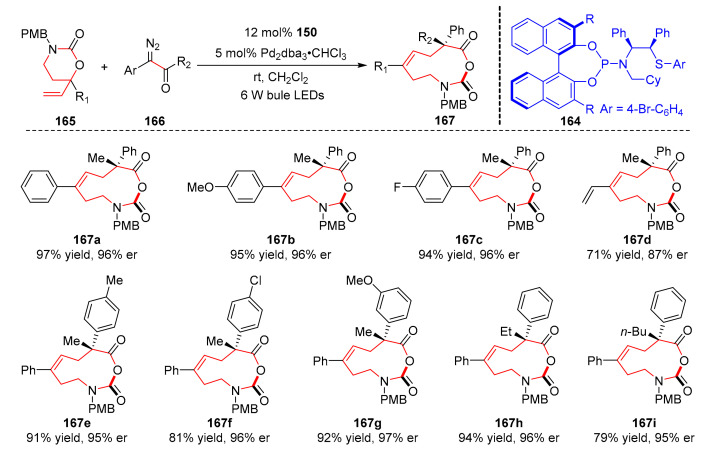
Palladium-catalyzed asymmetric [8+2] dipolar cycloadditions of vinyl carbamates and photogenerated ketenes.

Compared with mesocyclic molecules, chiral macrocyclic molecules are relatively rare, and the corresponding synthesis methods are not mature. In 2020, Xiao et al. reported palladium-catalyzed asymmetric [8+2] dipolar cycloadditions. In the presence of a chiral ligand (**164**) and Pd_2_(dba)_3_·CHCl_3_, vinyl carbamates (**165**) could react with photogenerated ketenes (**166**) to deliver 10-membered cycloadducts (**167**) upon the diffraction of blue LED light in excellent yields and enantioselectivity (up to 97% yield, up to 97% ee) (Scheme 36) [46]. A variety of vinyl carbamates bearing different aryl groups could afford desired cycloadducts with high er, and unsaturated vinyl group substituted with vinyl carbamate could be well tolerated in asymmetric [8+2] cycloaddition. Electronically different substituents at the phenyl ring of α-diazoketones showed excellent applicability; α-diazoketones with alkyl groups, such as methyl, ethyl, and *n*-butyl are also applicable in this reaction. However, 2-aryl or alkyl-substituted vinyl carbamates and 2-aryl or alkenyl-substituted α-diazoketones cannot participate in the reaction. This method is the first visible light induced asymmetric [8+2] cycloaddition reaction.

### 2.7. Enantioselective Formation of Multi-Ring by Visible Light Catalysis

**Scheme 37:**
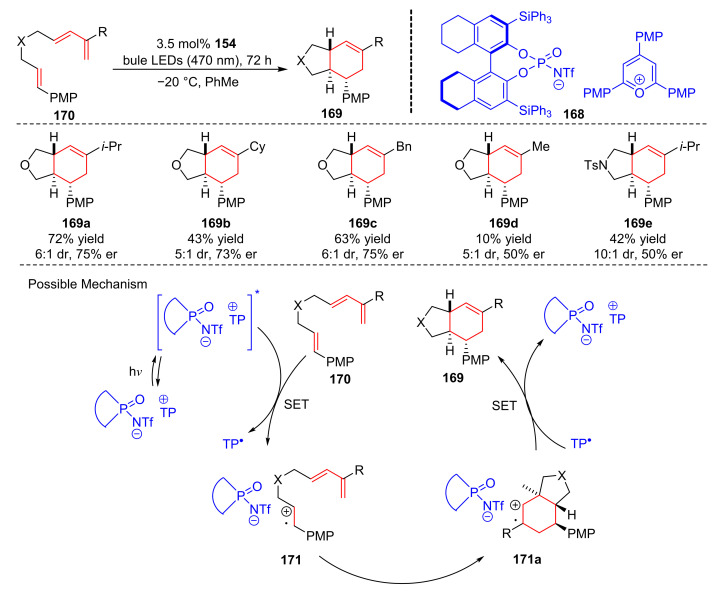
Asymmetric cation radical intramolecular Diels–Alder reaction of trienes.

Compared with traditional synthesis methods, photocatalytic synthesis of chiral polycyclic compounds is new and effective. In 2018, Nicewicz et al. reported an asymmetric cation radical intramolecular Diels–Alder reaction, utilizing an oxidizing pyrilium salt bearing a chiral N-triflyl phosphoramide anion (**168**) to synthesize cycloaddition products with bicyclic structure (**169**) from trienes (**170**) upon the diffraction of blue LED light (λ = 470 nm) in good yields and enantioselectivity (up to 72%, up to 75% er) (Scheme 37) [47]. Moreover, this reaction could also be used to yield [2.2.1]-bicycloheptenes. The proposed mechanism was conducted as follows. Electron-rich dienophile of substrates (**170**) underwent the one-electron oxidation by photoredox catalyst upon the diffraction of blue LED light to transform into radical intermediates (**171**), which gave radical intermediates (**171a**) via cyclization. One-electron reduction of intermediates (**171a**) furnished bicyclic products (**169**).

**Scheme 38:**
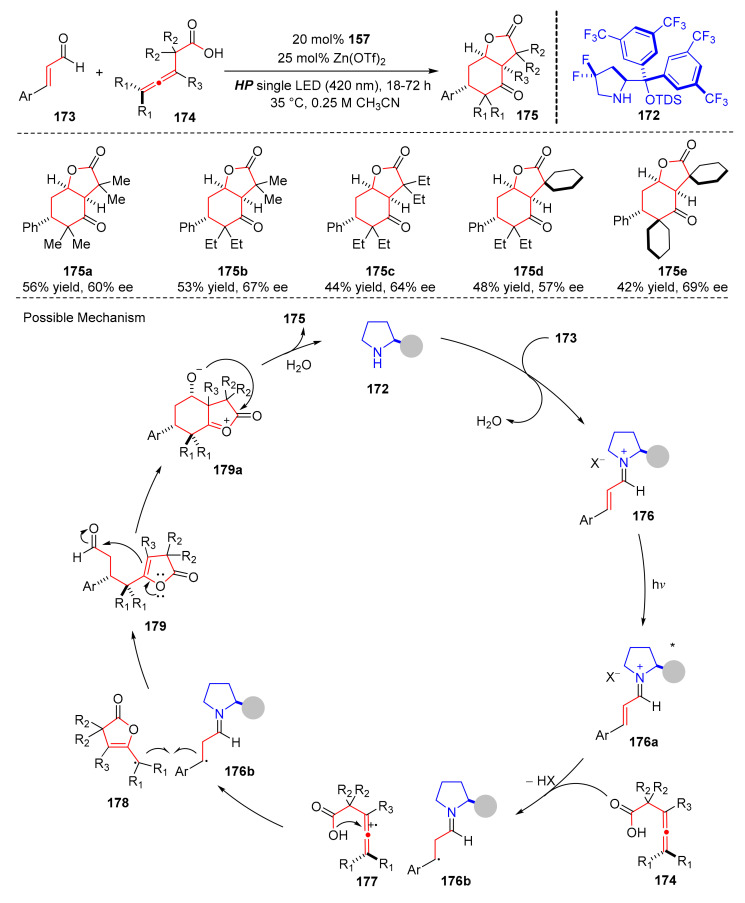
Enantioselective photocatalysis of α,β-unsaturated aldehydes and allenes.

In 2019, Melchiorre et al. reported an enantioselective photocatalysis which utilized chiral amine (**172**) as a photo-organocatalyst, and α,β-unsaturated aldehydes (**173**) reacted with allenes (**174**) to provide bicyclic lactones (**175**) upon the irradiation of single high-power (HP) LED light (λ = 420 nm) in good yields and enantioselectivity (up to 92% yield, up to 88% ee) (Scheme 38) [48]. Allenes bearing bulkier substituents showed better enantioselectivity. Moreover, the reaction could be also successfully applied to synthesize tetracyclic adducts. The proposed mechanism was conducted as follows. The reaction began with the condensation of the chiral amine catalyst (**172**) and a α,β-unsaturated aldehydes (**173**) to form chiral iminium ions (**176**), which was excited to give strong oxidants (**176a**). Allenes (**174**) underwent SET activation with (**176a**) to generate the chiral 5π-electron intermediates (**176b**) and the allene cation radicals (**177**), then a polar-radical-crossover addition of allene radical cations (**177**) could afford the tertiary radicals (**179**), which reacted with chiral 5π-electron intermediates (**176b**) to intermediates (**179**). An aldol-type cyclisation could occur in intermediates (**179**) to provide adducts (**179a**), which finally furnished bicyclic lactones (**175**) through acyl migration step and complete catalytic cycle.

**Scheme 39:**
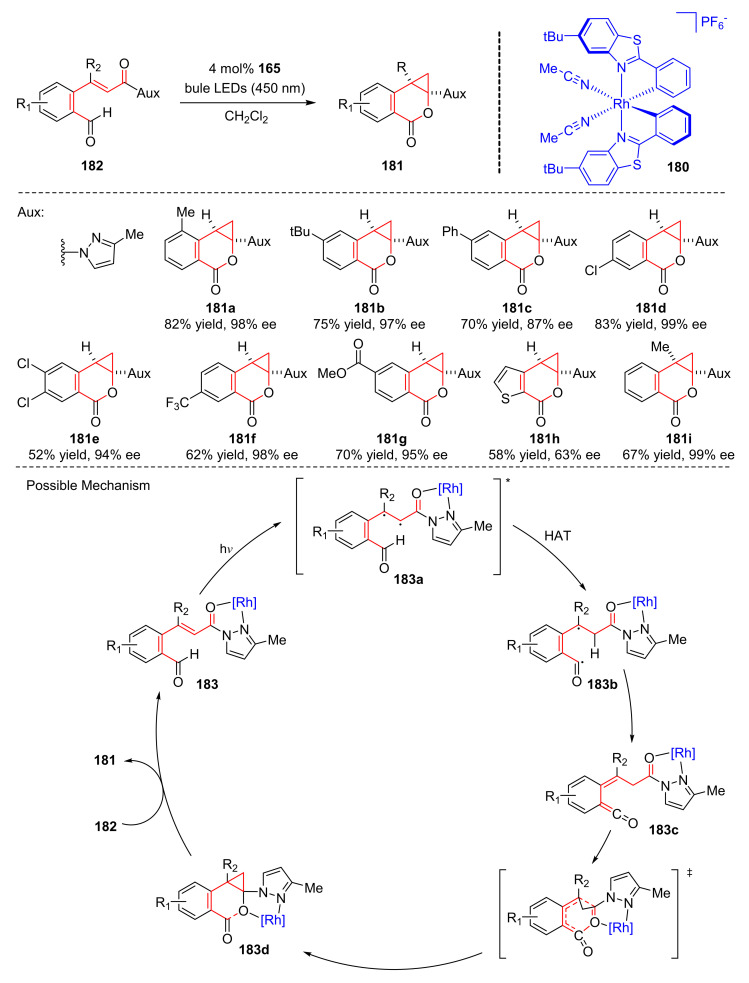
Asymmetric photocatalysis 3-(2-formylphenyl)-1-pyrazol-1-yl-propenones by intramolecular hydrogen-atom transfer.

In 2019, Meggers et al. used a bis-cyclometalated rhodium catalyst (**180**) to synthesize benzo[*d*]cyclopropa[*b*]pyranones (**181**) from 3-(2-formylphenyl)-1-pyrazol-1-yl-propenones (**182**) upon the irradiation of visible light (λ = 450 nm) in good yields and enantioselectivity (up to 93% yield and up to >99% ee) (Scheme 39) [49]. Different substituents at any position of the phenyl moiety, such as methyl, bulky, electron-withdrawing, and electron-donating substituents, were well tolerated. The proposed mechanism was conducted as follows. Substrates (**182**) initially could combine with a rhodium catalyst to form complex (**183**), complex (**183**) could transform into photoexcited complex (**183a**) in the presence of visible light, which underwent HAT to generate diradical intermediates (**183b**). Next, singlet-state ketenes (**183c**) went through intersystem crossing and hetero-Diels–Alder reaction to produce rhodium bound cyclopropanes (**183d**) which finally released desired products (**181**).

**Scheme 40:**
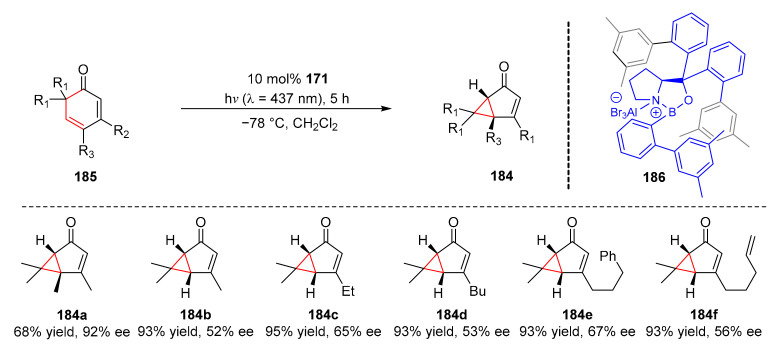
Enantioselective photochemical rearrangement of 2,4-cyclohexadienones.

In 2019, Bach et al. reported an enantioselective photochemical rearrangement to synthesize bicyclic ketones (**184**) from 2,4-cyclohexadienones (**185**) upon the irritation of visible light (λ = 437 nm) in good yields and high enantioselectivity (52–80% yield, 92–96% ee) with a chiral Lewis acid (**186**) as a catalyst (Scheme 40) [50]. A series of 3-alkyl substituted 2,4-cyclohexadienones could readily participate in rearrangement to obtain bicyclic ketones products.

**Scheme 41:**
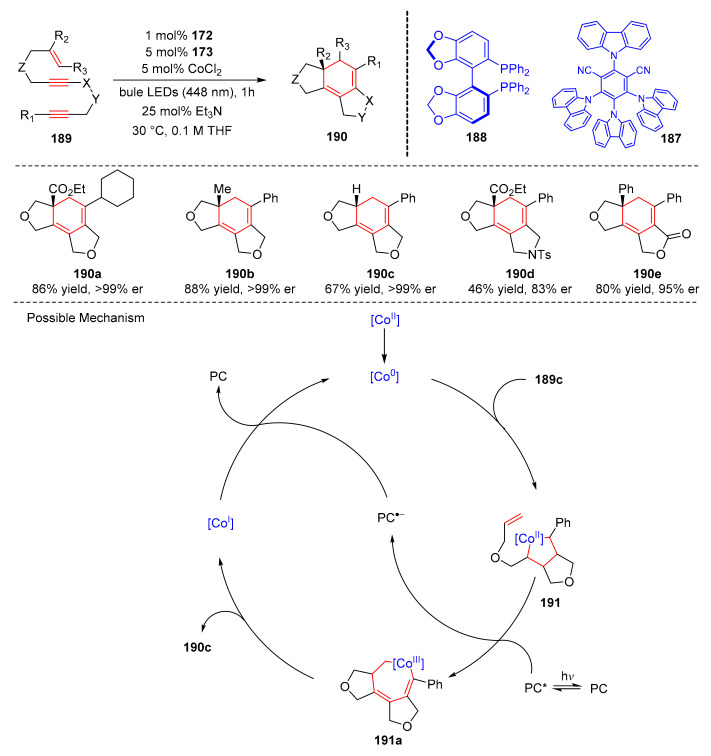
Enantioselective [2+2+2] cycloaddition of enediynes.

In 2021, Yamamoto et al. found an enantioselective [2+2+2] cycloaddition via dual cobalt and photoredox catalysis (**187**), with a chiral bisphosphine (**188**) as ligand. In the presence of bule light (λ = 448 nm), enediynes (**189**) could produce tricyclic cyclohexadienes bearing a quaternary bridgehead carbon (**190**) in good yields and enantioselectivity (up to 88% yield, up to >99% er) (Scheme 41) [51]. A variety of enediynes were well tolerated to obtain corresponding cyclohexadienes. The proposed mechanism was conducted as follows. Co^II^ precatalyst was reduced to Co^0^ by photoredox catalysis, substrate (**189c**) underwent the yne-yne coupling with Co^0^ catalyst to generate cobaltacyclopentadiene intermediate (**191**), which could give intermediate (**191a**) via photoinduced oxidation. Finally, intermediate (**191a**) were reduced to furnish product (**190c**) and the Co^I^ species, Co^I^ through photocatalyst-mediated reduction to restore Co^0^ and compete catalytic cycle.

## 3. Conclusions

In the past 5 years, enantioselective visible light catalysis has become an important strategy for the synthesis of chrial organic molecules. In this review, we have mainly summarized the new methods for the construction of chiral cyclic compounds via photo-induced transformation. The substrate applicability and mechanism of various methods are briefly described. It is worth noting that these photochemical synthesis methods provide a good supplement for the construction of polychiral and polycyclic compounds which are difficult, or even impossible, to synthesize with the previous methods. It can be predicted that photocatalysis will become a greener and more environmentally friendly synthesis method and will play an important role in the synthesis of a variety of corresponding chiral compounds, providing new ideas for the total synthesis of natural products and drugs.
